# An Assessment of Heavy Ion Irradiation Mutagenesis for Reverse Genetics in Wheat (*Triticum aestivum* L.)

**DOI:** 10.1371/journal.pone.0117369

**Published:** 2015-02-26

**Authors:** Timothy L. Fitzgerald, Jonathan J. Powell, Jiri Stiller, Terri L. Weese, Tomoko Abe, Guangyao Zhao, Jizeng Jia, C. Lynne McIntyre, Zhongyi Li, John M. Manners, Kemal Kazan

**Affiliations:** 1 Commonwealth Scientific and Industrial Research Organisation, Agriculture Flagship, Queensland Bioscience Precinct, 306 Carmody Rd, St Lucia, Brisbane, QLD, 4067, Australia; 2 Queensland Alliance for Agriculture and Food Innovation, University of Queensland, St. Lucia, QLD, 4072, Australia; 3 RIKEN Nishina Center for Accelerator-Based Science, 2-1 Hirosawa, Wako, Saitama, 351–0198, Japan; 4 National Key Facility for Crop Resources and Genetic Improvement, Institute of Crop Science, Chinese Academy of Agricultural Sciences, Beijing 100081, China; 5 Commonwealth Scientific and Industrial Research Organisation, Agriculture Flagship, Black Mountain Laboratories, Clunies Ross St, Acton, ACT, 2601, Australia; University of Guelph, CANADA

## Abstract

Reverse genetic techniques harnessing mutational approaches are powerful tools that can provide substantial insight into gene function in plants. However, as compared to diploid species, reverse genetic analyses in polyploid plants such as bread wheat can present substantial challenges associated with high levels of sequence and functional similarity amongst homoeologous loci. We previously developed a high-throughput method to identify deletions of genes within a physically mutagenized wheat population. Here we describe our efforts to combine multiple homoeologous deletions of three candidate disease susceptibility genes (*TaWRKY11*, *TaPFT1* and *TaPLDß1*). We were able to produce lines featuring homozygous deletions at two of the three homoeoloci for all genes, but this was dependent on the individual mutants used in crossing. Intriguingly, despite extensive efforts, viable lines possessing homozygous deletions at all three homoeoloci could not be produced for any of the candidate genes. To investigate deletion size as a possible reason for this phenomenon, we developed an amplicon sequencing approach based on synteny to *Brachypodium distachyon* to assess the size of the deletions removing one candidate gene (*TaPFT1*) in our mutants. These analyses revealed that genomic deletions removing the locus are relatively large, resulting in the loss of multiple additional genes. The implications of this work for the use of heavy ion mutagenesis for reverse genetic analyses in wheat are discussed.

## Introduction

Recent advances in high-throughput sequencing technologies have vastly increased understanding of gene content and variation within many plant species [1]. For some plants such as Arabidopsis (*Arabidopsis thaliana*) and rice (*Oryza sativa*), completed genome sequences are available and re-sequencing of numerous accessions has been performed [2], [3]. Even for crops with complex genomes, large quantities of sequence data have been produced and genome references of varying degrees of completeness are beginning to emerge [4], [5], [6]. Despite the power of these resources as platforms for functional genomics [7], [1] characterization of gene function clearly requires additional analyses. Gene inactivation is a powerful approach for the study of gene function, and has contributed substantially to our current knowledge in this area [8]. Furthermore, in instances where the loss of function of a gene is known or predicted to confer a desirable agronomic or quality phenotype, gene inactivation has potential to be used as a tool for crop improvement, e.g. [9].

In the model plant Arabidopsis, for most genes a T-DNA insertion and/or EMS mutant is publically available [10] allowing efficient study of the effect of loss of gene function. In most crop plants such comprehensive resources are not yet available and the study of loss-of-function mutants is not as simple. However TILLING approaches [11] are effective in identifying mutant gene variants in crops, some of which are non-functional. Traditionally, mutant populations for TILLING have been developed using chemical mutagens such as ethyl methanesulfonate (EMS) or sodium azide (NaN_3_), which predominantly produce point mutations with high frequency and random distribution within the genome [12]. Physical mutagens such as gamma rays, fast neutrons and heavy ions have also been used for plant mutagenesis and reverse genetic [13] strategies [14], [15]; these physical agents mainly induce genomic deletions. The size distribution of deletions varies with specific mutagen/plant species combinations, but is known to range from only individual base pairs to hundreds of kilobases [16]. Additionally, structural changes including inversions and translocations are relatively common as a result of physical mutagenesis [17].

Bread wheat (*Triticum aestivum*) is one of the most important agricultural commodities in the world, cultivated on the largest land area of any crop plant and providing 20 percent of human caloric intake globally [5]. Functional genomics has great potential to assist wheat improvement, however the wheat genome is notoriously challenging for functional analysis [4]. In addition to its extremely large size (~ 17 Gb; approximately 40 times the size of rice) and high percentage of repetitive elements (80–90%; [18], [19]) the bread wheat genome is hexaploid, possessing three ‘subgenomes’ designated ‘A’, ‘B’ and ‘D’. For most wheat genes, six homoeologous copies exist, two alleles from each of the subgenomes, which is assumed to lead to a high degree a functional redundancy [13].

Most likely due to its polyploid genome, wheat is extremely tolerant of mutation. For example, a mutation rate of ~ 1 in 32 kb has been reported within a chemically mutagenized TILLING population in bread wheat, compared to an average of ~ 1 in 400 kb in diploid plants [20]. While such high tolerance to mutation is desirable in the sense that ‘mutation saturation’ (i.e. production of a population statistically likely to contain a mutation in any given gene) can be achieved with relatively small population sizes [20], genomic redundancy can potentially present a substantial challenge for the production of loss-of-function mutants for a given gene. Theoretically, to achieve complete gene inactivation individual loss of function mutations of all six homoeologous gene copies would need to be combined in a single line. Since the seminal work of Slade *et al*. [21], reports of functional analysis of genes and/or plant improvement via TILLING in wheat are limited [13], although protocols specifically designed for TILLING in wheat have been reported [22]. It seems likely that difficulties (such as the absence of a detectable phenotype) associated with genetic redundancy may have contributed to this.

Previously, we reported the development of a method to facilitate high-throughput screening for homoeologous gene deletions within a mutant wheat population produced using heavy ion irradiation (HII) [15]. We demonstrated the application of the method to the identification of deletions of wheat homologues of several disease susceptibility genes [15] including *PFT1* [23] and *PLDß1* [24] characterized in model plants. Such susceptibility genes are seen as good candidates for this approach, as wheat inactivations may represent novel sources of disease resistance that have great potential to assist with crop improvement. However, given the hexaploid nature of the wheat genome, substantial additional development of genetic stocks is required to fully explore this strategy. Ideally, for a given wheat gene lines would be produced which feature 1. deletions of each of the individual homoeoloci; 2. all combinations of deletions of two of three homoeoloci (i.e. ‘AB’, ‘BD’, and ‘AD’ deletions); and 3. Deletions of all homoeoloci (i.e. ‘ABD’ deletions). Here we outline our attempts to create comprehensive deletion stocks for three candidate susceptibility genes in wheat using HII deletion mutants, and provide an assessment of the use of HII mutagenesis for reverse genetics in wheat.

## Results

### Identification of ‘primary’ Chara HII deletion mutants

Identification of mutant lines possessing homozygous deletions of individual homoeoloci (‘primary’ mutants) of the putative disease susceptibility genes *TaPLDß1* and *TaPFT1* in the wheat cultivar ‘Chara’ was described previously [15]. Here we also focussed on *TaWRKY11*, another putative disease susceptibility gene. In Arabidopsis, WRKY11 acts as a negative regulator of resistance to the hemibiotrophic bacterial pathogen *Pseudomonas syringae*. T-DNA mutants possessing inactivated WRKY11 demonstrate enhanced resistance to both virulent and avirulent strains of the pathogen [25]. Sequences of three putatively homoeologous copies (S1 Fig.) of a wheat homologue of Arabidopsis *WRKY11*, which share between 96–98% identity (S1 Fig.), were identified as described in Materials and Methods. A modified version of the method of Fitzgerald *et al*. [15] (see Materials and Methods) was then used to identify primary *TaWRKY11* deletion mutants. Previously, we observed an average frequency of candidate gene deletions within the HII population of approximately 0.3% (i.e. 3 independent deletions in the same gene in 1000 lines) [15]. DNA from 1728 M2 Chara HII mutants was initially screened to identify putative *TaWRKY11* mutants. Re-screening of M3 plants obtained from putative mutants confirmed deletions in six lines; four A-homoeologue (*TaWRKY11-A*) deletions and two B-homoeologue (*TaWRKY11-B*) deletions (Tables A and B in S1 File). However, no confirmed D-homoeologue (*TaWRKY11-D*) mutants could be identified at the M3 generation. Subsequently an additional 768 DNA samples were screened and a single *TaWRKY11-D* deletion was identified at the M3 generation (Tables A and B in S1 File).

### Chromosomal mapping of candidate genes

To further assess whether the highly homologous copies targeted by our screening method are, in fact, located on the homoeologous chromosomes of the A, B and D sub-genomes, respectively, the Chinese Spring nullisomic-tetrasomic lines [26], which lack individual homoeologous chromosome pairs, were screened using the method of Fitzgerald *et al*. [15]. The chromosomal locations of the *TaPFT1* sequences targeted by each of the fluorescently-labelled probes were previously determined to be on the group 5 chromosomes 5A, 5B, and 5D [15]. Nullisomic-tetrasomic lines lacking the 1A, 1B and 1D homoeologous chromosome pairs showed inefficient/absent fluorescence from the PLDB1prbFAM, PLDB1prbVIC and PLDB1prbNED probes, respectively. This indicates that the *TaPLDß1* sequences are located on these homoeologous chromosomes (S2 Fig.; Table C in S1File). For *TaWRKY11*, nullisomic-tetrasomic lines lacking the 2B and 2D chromosomes showed inefficient/absent fluorescence from the WRKY11prbNED and WRKY11prbFAM probes, respectively, indicating that *TaWRKY11* homoeologue sequences are located on these chromosomes (S3 Fig.; Table C in S1 File). For the chromosome 2A series, only DNA from monosomic lines (possessing a single 2A chromosome) was present in the collection available to us and thus the location of a *TaWRKY11* homoeologue on 2A could not be confirmed using this approach, as the probe-based screening method produces similar fluorescence from a single copy of a target sequence (as present on a monosomic chromosome) as two copies [15].

With the recent availability of the ‘Whole Chromosome Survey Sequence’ or ‘CSS’, (International Wheat Genome Sequencing Consortium; http://plants.ensembl.org/info/website/ftp/index.html; as published in [27]), the chromosomal location of *TaPFT1*, *TaPLDß1* and *TaWRKY11* was assessed *in silico* by a MEGABLAST search of all chromosome-arm specific sequence data with gene sequence used for the design of the high-throughput deletion screening assays. Results of this analysis complemented those obtained from screening of the nullisomic-tetrasomic accessions and are described in S2 File.

### Combining multiple homoeologous deletions

To overcome possible redundancy conferred by homoeologous copies of our candidate genes, attempts were made to produce lines with homozygous deletions of two homoeoloci (one intact homoeologous locus remaining; ‘tetras’) and three homoeoloci (all homoeologues removed; ‘hexas’). Viable tetras were produced for all genes (Table D in S1 File), but as outlined in detail below this was dependent on the individual mutants used in crossing. However, viable hexas could not be produced for any of our candidate genes despite extensive efforts.

For each candidate gene, crosses were made between mutants possessing homozygous deletions of different homoeoloci, and F2 progeny was screened to identify lines featuring homozygous deletions of multiple homoeoloci. As stated above, the high-throughput screening method used does not distinguish between hemizygous individuals lacking one allele at a given homoeolocus and homozygous wild type individuals possessing both alleles. Rather, individuals homozygous for the deletion of a specific homoeolocus (i.e. with both homoeoalleles deleted) are detected [15]. The expected genotypic ratios as detected by the probe screen of F2 progeny of the crosses performed for this study are outlined in Table E in S1 File. Details of crosses performed, progeny screened and the probability of the observed genotypic ratios in the progeny of a given cross based on a Chi-square analysis [28] are presented in Table 1 and Table 2.

**Table 1:** Overview of attempts to create ‘tetras’ for *TaPFT1*, *TaPLDB1* and *TaWRKY11*.

Gene Name	A parent	B parent	D parent	Index of expected ratios‡	No. of F2 progeny screened	A-deletion	B-deletion	D-deletion	AB-deletion	AD-deletion	BD-deletion
*TaPFT1*	*tapft1–616-a*	*tapft1–157-b*	-	1	316	55	51	-	0**	-	-
*TaPFT1*	*tapft1–616-a*	-	*tapft1–723-d*	2	164	21	-	18**	-	0**	-
*TaPFT1*	*tapft1–85-a*	-	*tapft1–734-d*	2	85	14	-	19	-	9	-
*TaPFT1*	-	*tapft1–157-b*	*tapft1–723-d*	3	124	-	17	17	-	-	0**
*TaPFT1*	*tapft1–85-a*	*tapft1–157-b*	-	1	124	22	19	-	0**	-	-
*TaPFT1*	*tapft1–616-a*	-	*tapft1–757-d*	2	83	18	-	4**	-	0*	-
*TaPFT1*	*tapft1–616-a*	-	*tapft1–734-d*	2	39	5	-	2*	-	2	-
*TaPFT1*	-	*tapft1–157-b*	*tapft1–757-d*	3	192	-	41	23*	-	-	3**
*TaPFT1*	-	*tapft1–157-b*	*tapft1–734-d*	3	110	-	15	9**	-	-	7
*TaPLDß1*	*tapldß1–690-a*	*tapldß1–150-b*	-	1	75	8	16	-	0*	-	-
*TaPLDß1*	*tapldß1–781-a*	*tapldß1–855-b*	-	1	48	3*	10	-	4	-	-
*TaPLDß1*	*tapldß1–334-a*	*tapldß1–855-b*	-	1	40	4	2*	-	1	-	-
*TaPLDß1*	*tapldß1–208-a*	*tapldß1–855-b*	-	1	48	8	12	-	0	-	-
*TaPLDß1*	*tapldß1–781-a*	-	*tapldß1–934-d*	2	134	31	-	18	-	9	-
*TaPLDß1*	*tapldß1–334-a*	-	*tapldß1–147-d*	2	94	24	-	17	-	5	-
*TaPLDß1*	*tapldß1–781-a*	-	*tapldß1–147-d*	2	42	4	-	9	-	5	-
*TaPLDß1*	-	*tapldß1–855-b*	*tapldß1–147-d*	3	92	-	21	12	-	-	7
*TaPLDß1*	-	*tapldß1–150-b*	*tapldß1–934-d*	3	32	-	4	6	-	-	2
*TaWRKY11*	*tawrky11–208-a*	*tawrky11–743-b*	-	1	104	16	22	-	3	-	-
*TaWRKY11*	*tawrky11–286-a*	*tawrky11–87-b*	-	1	51	11	10	-	2	-	-
*TaWRKY11*	*tawrky11–227-a*	*tawrky11–87-b*	-	1	76	4**	11	-	0*	-	-
*TaWRKY11*	*tawrky11–417-a*	*tawrky11–87-b*	-	1	44	4	6	-	3	-	-
*TaWRKY11*	*tawrky11–227-a*	-	*tawrky11–300-d*	2	48	4	-	4	-	1	-
*TaWRKY11*	*tawrky11–417-a*	-	*tawrky11–300-d*	2	48	8	-	8	-	6	-
*TaWRKY11*	*tawrky11–286-a*	-	*tawrky11–300-d*	2	48	5	-	6	-	4	-

The number of progeny screened (F2 progeny) and the number of progeny identified with each expected deletion genotype (e.g. A-deletion) is given. ‘-’ indicates that a given parent was not used in the cross, or a given deletion was not expected in the progeny. Asterisks indicate significantly different observed frequencies of a deletion genotype compared to expectations;

* indicates P < 0.05;

** indicates P < 0.01.

Where a deletion genotype was not identified and this was significantly different from expectations, this is in bold.

‡ ‘Index’ refers to the expected genotypic ratio within F2 progeny of crosses performed as given in Table E in S1 File.

**Table 2:** Overview of attempts to create ‘hexas’ for *TaPLDß1* and *TaWRKY11*.

Gene	A parent	B parent	D parent	Index of expected ratios‡	No. of F2 progeny screened	AB-deletion	AD-deletion	BD-deletion	ABD-deletion
*TaPLDß1*	*tapldß1–781-a*	*tapldß1–855-b*	*tapldß1–934-d; tapldß1–147-d*	4	79	-	13	19	5
*TaPLDß1*	*tapldß1–334-a*	*tapldß1–855-b*	*tapldß1–147-d*	4	64	-	9	9	4
*TaWRKY11*	*tawrky11–417-a*	*tawrky11–87-b*	*tawrky11–300-d*	5	252	8	9	0**	0*
*TaWRKY11*	*tawrky11–286-a*	*tawrky11–87-b*	*tawrky11–300-d*	5	283	7	2	0**	0*

Number of progeny screened (F2 progeny) and number of progeny identified with expected tetra and hexa genotypes (frequency of primary mutants is not shown; these were similar to expected for all crosses.

‘-’ indicates that a given parent was not used in the cross, or a given deletion was not expected in the progeny. N.B. for *TaPLDß1* A-D tetras were crossed with B-D tetras to produce hexas, and thus all progeny were expected to possess a D deletion. Therefore AB-deletion lines (possessing deletions of A and B, but with D intact) were not expected within the progeny.

Asterisks indicate significantly different observed frequencies of a deletion genotype compared to expectations;

* indicates P < 0.05;

** indicates P < 0.01. Where a deletion genotype was not identified and this was significantly different from expectations, this is in bold.

‡ ‘Index’ refers to the expected genotypic ratio within F2 progeny of crosses performed as given in Table E in S1 File.

**Fig 1:**
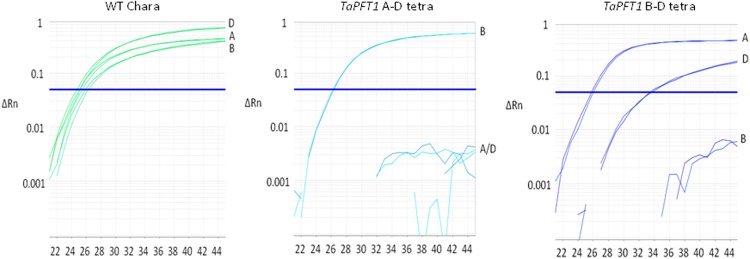
Fluorescence profiles of *TaPFT1* tetras detected using the high-throughput screening method. Note that for B-D tetras some off-target fluorescence from the D-specific probe was routinely observed. However the signal could be clearly distinguished from that of the intact D target. Furthermore, the absence of D target in B-D tetras was confirmed using an independent CAPS assay (see S2 File and S10 Fig.). All images incorporate duplicate reactions and duplicate signals from each probe.

For *TaPFT1*, nine independent mutant combinations (two A-B; four A-D; and three B-D) were used to produce tetras. 1237 F2 progeny were screened and A-D and B-D tetras were identified and confirmed at the F3 generation (Table 1; e.g. Fig. 1). However, tetras could not be obtained for five of the mutant combinations, including all A × B attempts; in all cases this was significantly different from statistical expectations (Table 2). Since all A and B homoeologue deletion mutants were incompatible, the production of *TaPFT1* hexas appeared unfeasible and this was not attempted.

For *TaWRKY11*, seven independent mutant combinations (four A-B; and three A-D) were used to produce tetras. 419 F2 progeny were screened and A-B and A-D tetras were identified and confirmed at the F3 stage (e.g. S4 Fig.). Tetras could not be produced from one mutant combination (*tawrky11–227-a* × *tawrky11–87-b*) and this was significantly different from statistical expectations (Table 1). A-B tetras produced from two different primary mutant combinations (*tawrky11–417-a* × *tawrky11–87-b*; and *tawrky11–286-a* × *tawrky11–87-b*) were then crossed with *tawrky11–300-d* to produce hexas (Table 2). Although A-B and A-D tetras were produced at similar to expected ratios, B-D tetras were not observed within the F2 progeny screened; again this was significantly different from expectations based on Chi-square analysis (Table 2). Similarly, hexas could not be obtained despite screening of 535 F2 progeny, and Chi-square analysis indicated that this was a significant deviation from expectations (Table 2).

For *TaPLDß1*, nine independent mutant combinations (four ‘A-B’; three ‘A-D’; and two ‘B-D’) were used in crossing experiments to produce tetras (Table 1). A total of 605 F2 progeny were screened and A-B, A-D, and B-D tetras were identified and confirmed at the F3 generation (e.g. S5 Fig.). For two A-B mutant combinations, no tetras were detected and for one combination (*tapldß1–690-a* × *tapldß1–150-b*), this was significantly different from expectations based on number of progeny screened (Table 1). Crosses were then made between A-D tetras from two different mutant combinations (*tapldß1–334-a* × *tapldß1–147-d*; and *tapldß1–781-a* × *tapldß1–934-d*), and B-D tetras from *tapldß1–147-d* × *tapldß1–855-b* and F2 progeny of these crosses was screened to identify hexas. All mutant combinations produced hexas at similar to expected frequencies (Table 2). However, all hexas produced were sterile (S6 Fig.). Artificial fertilization of the lines with wild-type pollen produced viable seed, indicating that the combination of deletions at the three *TaPLDß1* homoeoloci induced male sterility (S6 Fig.).

Genotypes of *TaPFT1* and *TaWRKY11* tetras, and *TaPLDß1* hexas were independently confirmed using agarose gel-based assays as outlined in S2 File and S10 Fig.

### Assessment of deletion size in wheat mutants using synteny to *Brachypodium distachyon*

Heavy ion irradiation is known to be capable of producing relatively large deletions even in diploid plant species e.g. [16]. We hypothesized that potentially large deletion sizes may contribute to the inability to ‘stack’ multiple homoeologous deletions in our wheat mutants. To explore this hypothesis, we developed an amplicon sequencing approach based on synteny to *Brachypodium distachyon* to investigate the size of the deletions removing *TaPFT1* (Materials and Methods).

**Fig 2:**
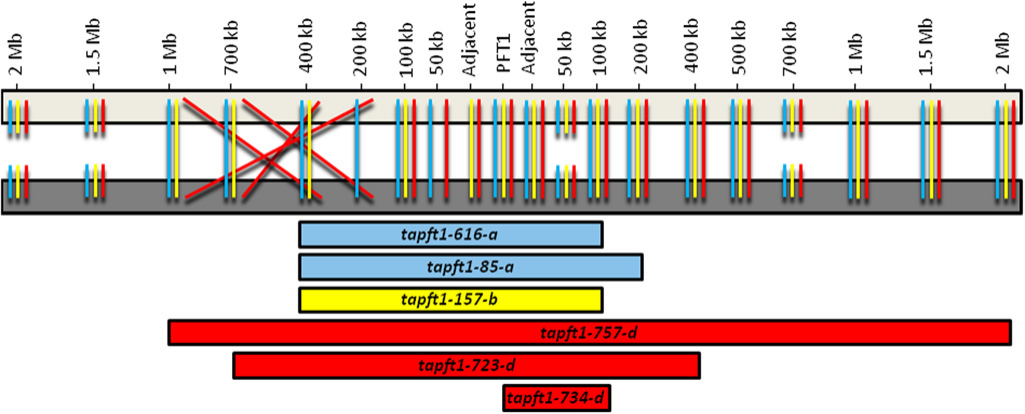
Overview of synteny of genes flanking *PFT1* in wheat and Brachypodium, and deletions removing *TaPFT1* in wheat mutants. Light grey bar represents Brachypodium *BdPFT1* region on chromosome 4; dark grey bar represents wheat *TaPFT1* region on chromosome 5 series. Left of *PFT1* is treated as ‘Up’, right of *PFT1* is treated as ‘Down’. Blue, yellow, and red lines demonstrate relative gene order in Brachypodium and the A, B, and D homoeologous wheat chromosomes, respectively. Where a line is absent (e.g. for 50 kb Up) data was insufficient to determine synteny of this gene for that subgenome. Broken lines (e.g. 50 kb Down) indicate loss of synteny, i.e. a wheat homoeologue was not identified within the syntenic region. Blue, yellow, and red bars represent deletions removing *TaPFT1* in A, B, and D primary mutants, respectively. Bars extend to the gene furthest from *PFT1* shown to be deleted in a *TaPFT1* mutant; deletions may extend beyond this region until the location of the first gene shown to be intact (e.g. the size of the Brachypodium region syntenic to the deletion in *tapft1–616-a* falls in the range of 400 kb Up—100 kb Down [500 kb], to 700 kb Up—200 kb Down [900 kb]).

For this approach, a putative *BdPFT1* homologue was identified on Brachypodium chromosome 4. Wheat homologues of 20 genes flanking *BdPFT1* at regular intervals were identified across a region of ~ 4 Mb (spanning locations ~ 2Mb from *BdPFT1* in opposite chromosomal directions; we use ‘Up’ to refer to the direction towards the distal end of the short arm of chromosome 4) (Table 3). Fragments of these genes were then PCR amplified from control samples and all *TaPFT1* deletion mutants used for this study, and were sequenced using the Ion Torrent platform. One fragment was excluded from analysis due to poor technical reproducibility amongst wild type controls. The remaining 19 fragments were assessed for evidence of a deleted homoeologue based on the absence of SNPs relative to wild type samples. Chromosome 5 series nullisomic-tetrasomic lines, previously demonstrated to lack A, B, and D homoeologues of *TaPFT1* [15] were incorporated as controls to assist with the identification of homoeologue-specific SNPs. For four genes, no homoeologue-specific SNPs could be identified by comparison of wild type samples with nullisomic-tetrasomic controls, suggesting a translocation of these genes in wheat relative to Brachypodium. For the remaining genes, deletion patterns in mutant and control samples are presented in Table F in S1 File and an overview of deletion sizes removing *TaPFT1* in the mutant lines, relative to the syntenic Brachypodium region, is provided in Fig. 2. Our analysis suggests that *TaPFT1* has been removed by deletions corresponding to syntenic regions in Brachypodium from less than 200 kb, to greater than 2 Mb in these mutants.

**Table 3:** Genes targeted for the amplicon sequencing approach to assess the size of deletions removing *TaPFT1* in HIB mutants.

Brachypodium gene	Location relative to BdPFT1	Wheat EST
Bradi4g25620 *	Up 2 Mb	CJ633275
Bradi4g26150 *	Up 1.5 Mb	CK209311
Bradi4g26877	Up 1 Mb	CK208423
Bradi4g27117	Up 700 kb	CD916847
Bradi4g27270 **	Up 500 kb	CD939242
Bradi4g27334	Up 400 kb	CJ874674
Bradi4g27490	Up 200 kb	CK162425
Bradi4g27607	Up 100 kb	HX155210
Bradi4g27720	Up 50 kb	CJ547672
Bradi4g27740	Up adjacent	TA96658_4565
Bradi4g27760	Down adjacent	EU714979
Bradi4g27777 *	Down 50 kb	CJ782327
Bradi4g27810	Down 100 kb	HX128956
Bradi4g27880	Down 200 kb	CJ617348
Bradi4g28040	Down 400 kb	CJ825949
Bradi4g28150	Down 500 kb	CJ690351
Bradi4g28310 *	Down 700 kb	CJ725966
Bradi4g28580	Down 1 Mb	BQ161472
Bradi4g29060	Down 1.5 Mb	CJ675064
Bradi4g29547	Down 2 Mb	CJ534934

* Based on amplicon sequencing in conjunction with *in silico* assessment using the CSS sequences, synteny between wheat and Brachypodium appears to have been lost for these genes.

** Based on inconsistency of SNP patterns in wild type control samples, the wheat homologue of this gene was excluded from further analysis.

With the recent availability of the CSS, as outlined above, we performed an *in silico* analysis of the synteny between Brachypodium and wheat for the genes assessed using this amplicon sequence approach (Materials and Methods). This analysis indicated that synteny was likely to be conserved for 14 genes, while for three genes synteny was likely to be lost (Table G in S1 File). For the remaining two genes the results were inconclusive (Table G in S1 File). For 13 genes for which synteny appeared conserved based on our amplicon sequencing analysis, *in silico* analysis was in agreement; for the remaining gene (Bradi4g26877) *in silico* analysis was inconclusive (Table F in S1 File). Likewise, of the four genes that appeared translocated based on our amplicon sequencing analysis, three were predicted as translocated while results for the fourth (Bradi4g27777) were inconclusive (Table G in S1 File). The high degree of agreement between the amplicon sequencing and *in silico* analyses is indicative of technical reliability of both approaches.

Based on the deletion patterns of the genes analysed (Table F in S1 File), a rearrangement of genes on chromosome 5D relative to Brachypodium is consistently predicted from the mutants *tapft1–723-d* and *tapft1–757-d*. The wheat homologues of the genes Bradi4g27490, Bradi4g27340, Bradi4g27120, and Bradi26880 located ~ 200 kb, ~ 400 kb, ~ 700 kb, and ~ 1Mb up from *BdPFT1*, respectively appear to have re-ordered with respect to the location of the genes in Brachypodium, as outlined in Fig. 2. The loss of microsynteny within regions that are broadly syntenic is known to be common in plants including cereals [29], and disrupted microsynteny has previously been observed between wheat and *Brachypodium* spp. [30], [31], [32]. Thus, this observation is not unexpected.

Together, our *in vitro* and *in silico* analyses suggested that for eight of 19 genes assessed in this region, there has been some degree of rearrangement in wheat relative to Brachypodium. Therefore, we expanded *in silico* analysis to gain further insight into the conservation of synteny within the region targeted by this approach (Materials and Methods); results are presented in S3 File. Furthermore, we complemented this comparative analysis by retrieving information on the putative functions of these Brachypodium-wheat homologues (Materials and Methods; S3 File). This analysis suggests that for the region 1 Mb up to 2 Mb down from *BdPFT1*, synteny is broadly conserved between wheat and Brachypodium. However, for the region 1 Mb to 2 Mb up, macrosynteny appears to have been lost, with sequence for the vast majority of wheat homologues of genes in this region not identified on the group 5 chromosome long arms (S3 File). For most of these genes, best matches were identified in the 4AL, 4BS, and 4DS regions, respectively (S2 File; S6 File).

Finally, we used information on the conservation of synteny in this region in conjunction with the draft genomes of *T*. *urartu* and *Aegilops tauschii*, which represent the most complete wheat subgenome assemblies currently available, to compare the physical size of the *BdPFT1* and *TaPFT1* regions. For *T*. *urartu* and *A*. *tauschii*, sequence was obtained for all unique scaffolds harbouring genes for which synteny appeared conserved between wheat and Brachypodium (Materials and Methods). The total size of these scaffolds was 12291605 and 11145364 bp for *T*. *urartu* and *A*. *tauschii*, respectively.

## Discussion

Reverse genetic approaches offer great promise for gene function analyses in crop plants. However, such analyses are technically challenging in plants with highly redundant genomes [13]. In this study, we have assessed the potential of a population developed using heavy ion irradiation-mediated mutagenesis for reverse genetics in wheat using three candidate disease susceptibility genes.

Extensive efforts to combine multiple homoeologous deletions using newly identified *TaWRKY11* mutants and previously identified *TaPLDß1* and *TaPFT1* mutants [15] were undertaken. For this purpose, screening of F2 progeny from crosses involving multiple mutant combinations (Tables 1 and 2) was performed using our previously developed high-throughput method [15]. For all genes, tetra mutants (possessing homozygous deletions at two homoeoloci; lacking four of six homoeoalleles) could be produced, although this was dependent on the specific homoeologue and/or mutant accessions being combined. However, viable hexa mutants (lacking all of the six homoeoalleles) could not be produced for any of our candidate genes.

Substantial screening of the HII population used in this study for deletions of candidate genes has been performed, as reported here and in [15]. These efforts suggest that deletions are relatively evenly distributed; in the order of 1 in 1000 mutant lines have been consistently observed to possess a homozygous deletion at a specific homoeolocus. However, while the distribution of mutations induced by chemical mutagenesis is known to be essentially random [12], this has not been definitively established for heavy ion irradiation mutagenesis. Thus, it remains possible that there are genomic regions more susceptible to mutation via HII, which could potentially lead to incompatibility between mutant lines due to deletions at untargeted loci. Nevertheless, unless such regions were tightly linked to the targeted loci, this would not specifically affect the stacking of homoeologous deletions, and thus by far the greatest likelihood is that the incompatibilities observed in this study are due to the targeted homoeoloci themselves. It remains unclear, however, whether the cause of this incompatibility is 1. stacking of deletions of the targeted candidate genes; 2. stacking of deletions of another gene at the targeted homoeoloci; or 3. a cumulative effect of stacking of deletions of multiple genes at the targeted homoeoloci. The inactivation of homologues of our candidate genes is tolerated in diploid Arabidopsis, so the latter two possibilities may be more likely.

Our results indicate that relatively large deletions within the HII population may have contributed to the observed incompatibilities. Nevertheless, these results are quite surprising given that wheat is known to tolerate the loss of very large amounts of genetic material. For example, the ditelosomic series [33] contains viable lines lacking whole chromosome arms. Therefore, it appears that stacking of deletions at multiple homoeoloci may be comparatively poorly tolerated in wheat relative to deletions restricted to an individual subgenome.

For *TaPFT1*, screening to produce tetra mutants demonstrated that all combinations of *TaPFT1-A* and *TaPFT1-B* deletion mutants used for this study were incompatible (Table 1) and thus the production of *TaPFT1* hexa mutants was not attempted. Similarly, for *TaWRKY11*, we were not able to identify hexa mutants or *TaWRKY11* B-D tetra mutants (Table 2). These results suggest that there is strong negative selection against, or resultant lethality from the combination of deletions used in our attempts to produce *TaPFT1* A-B tetras, and *TaWRKY11* B-D tetras.

For *TaPLDß1*, the majority of mutant combinations resulted in viable tetras (Table 1). Additionally, hexas were detected at similar to expected frequencies within F2 progeny from both of two crosses incorporating distinct primary mutant combinations (Table 2). However, all mutants confirmed as hexas were male-sterile (S6 Fig.). This indicates that the combination of deletions at the three *TaPLDß1* homoeoloci within the mutants used for these crosses is incompatible with fertile pollen production. While it is possible that deletion of the targeted *TaPLDß1* locus is responsible for this sterility, down-regulation/inactivation of *PLDß1* does not appear to affect fertility in rice [24] or Arabidopsis [34], suggesting that other unknown genes, or a combination of the deletion of multiple genes, is a more likely explanation.

To gain insight into the size of deletions in the mutants identified for this study, we applied an amplicon sequencing approach based on Brachypodium synteny. The intactness of wheat homologues of genes flanking *BdPFT1* in a region spanning ~ 4 Mb was assessed. Our results suggest that deletion sizes corresponding to syntenic regions in Brachypodium from 100–200 kb, to greater than 2 Mb, removed *TaPFT1* in the mutants used for this study (Fig. 2). Based on these results in conjunction with *in silico* analysis using the recently released hexaploid wheat Chromosomal Survey Sequences [27], we identified genes in this region for which there appeared to be a loss of microsynteny between Brachypodium and wheat (S3 File). Interestingly, we also identified a putative breakpoint of macrosynteny between Brachypodium and wheat which occurred ~ 1 Mb from *BdPFT1* towards the distal end of the short arm (‘Up’) of Brachypodium chromosome 4 (S3 File).

In addition to polyploidization, the wheat genome has undergone substantial expansion due to the proliferation of transposable elements. Although the expansion of the wheat genome is not uniform [35], it could be expected that the physical size of syntenic regions would generally be larger in wheat than in Brachypodium. To compare the physical size of the regions flanking *PFT1* in Brachypodium and wheat, we obtained scaffolds from the draft genomes of *T*. *urartu* and *A*. *tauschii* that harboured homologues of genes flanking *BdPFT1* for which synteny appeared conserved. The total length of these scaffolds was ~ 11.1 Mb and ~ 12.3 Mb for *T*. *urartu* and *A*. *tauschii* genomes, respectively; approximately 3x the physical size of the region in Brachypodium. However, these estimates are likely to be conservative. Firstly, as indicated above, we detected a loss of macrosynteny from ~ 1 Mb up from *BdPFT1*, and thus scaffolds containing most genes beyond that region were not incorporated in these totals. Secondly, within the macrosyntenic region from ~ 1 Mb up to ~ 2 Mb down from *BdPFT1*, we excluded genes for which it appears that microsynteny has been lost between Brachypodium and wheat, but just as there appear to be genes in Brachypodium that are not present in wheat in this region, it could be expected that there are genes in wheat that are not present in Brachypodium; regions containing such genes could not be captured by our analysis. Thirdly, the *T*. *urartu* and *A*. *tauschii* genomes are incompletely assembled and thus there is likely to be additional sequence within this region in these genomes that is not captured within the obtained scaffolds. Nevertheless, even based on this conservative analysis, it appears that substantial genomic expansion has occurred in wheat relative to Brachypodium in the targeted region. This suggests that the physical size of the deletions removing *TaPFT1* in our wheat mutants is considerably larger than the syntenic Brachypodium regions.

For two of the genes for which macrosynteny appears conserved between Brachypodium and wheat within the region of *TaPFT1*, homologues in Arabidopsis have been previously characterised as necessary for embryo development or fertility (S3 File). Polymorphisms in At3g20780 (homologue to Bradi4g27057) encoding a DNA topoisomerase 6 subunit b are responsible for harlequin (dwarf, skotomorphogenic and sterile) phenotypes [36], and Arabidopsis mutants homozygous for T-DNA insertion within At4g29170 (homologue to Bradi4g27110) encoding a meiotic nuclear division protein 1 homolog are sterile [37]. However, our analysis suggests that these genes are located between ~ 700 kb and ~ 1 Mb up from *TaPFT1*, which is outside of the regions removed in the *TaPFT1-A* and *TaPFT1-B* deletion mutants used in this study (Fig. 2), suggesting that these genes are unlikely to be responsible for the incompatibilities for deletion stacking observed here.

An additional gene located between ~ 200 kb and ~ 400 kb up from *TaPFT1* (within the region found to be incompatible for deletion stacking) is a homologue of Arabidopsis ‘embryo defective 1381 protein’ (At2g31340), mutations of which reportedly lead to developmental arrest of embryos (https://www.arabidopsis.org/servlets/TairObject?name=AT2G31340&type=locus). It therefore appears plausible that this gene may have contributed to the observed incompatibilities. Nevertheless, there may be other wheat genes in this region that have not been identified by our comparative analysis using Brachypodium. Additional research is clearly required to definitively determine the factor(s) responsible for the incompatibilities observed here, not only for stacking of *TaPFT1* deletions, but also for *TaWRKY11* and *TaPLDß1*.

Although the number of mutants with varying deletion sizes used for this study is relatively small, limiting our ability to make firm conclusions about correlations between deletion size and the ability to combine homoeologous deletions, our data is suggestive of a relationship. We observed that the *TaPFT1* deletion mutant *tapft1–734-d* was most effective for the production of tetras, with tetras produced from all crosses using this accession (Table 2), and based on our assessment of deletion size *tapft1–734-d* possessed the smallest deletion of all mutants, restricted to a size syntenic to a region < 200 kb in Brachypodium (Fig. 2; Table F in S1 File). If it is generally the case that smaller deletions are easier to combine, the ability to optimize mutation dosage to minimize deletion size would be highly desirable for future reverse genetic approaches using heavy ion mutagenesis.

Several studies have explored the use of heavy ion mutagenesis in the model plant Arabidopsis. This has provided preliminary evidence that the nature of mutations induced can vary based on the conditions of treatment used. For heavy ion mutagenesis, parameters that can be adjusted include the specific ion used, the quantity of ionizing radiation (e.g. as measured in Gray; Gy), and the linear energy transfer (LET) of the particles. Based on a comparison of two studies, substantially higher LET using carbon ions resulted in more frequent large deletions with smaller differences in LET resulting in similar patterns of mutation [16], [38]. A further complicating factor is the degree to which induced mutations are transmitted to offspring. Naito *et al*. [39] observed that HII induced a range of deletion sizes at two loci in Arabidopsis. Generally, the largest deletions (sizes of up to > 6 Mb) were not transmitted to progeny, moderate sized deletions were transmitted in the heterozygous state only, and small deletions/point mutations were transmitted normally.

Detailed information on the effect of adjusting HII dosage parameters on the pattern of induced mutations is lacking in plants generally. Further study in this area may allow for the optimization of HII to induce a mutation spectrum in wheat that is more conducive to reverse genetics, as posited above. However, given that wheat is highly tolerant to large deletions, the information obtained from other species may not be predictive; e.g. large deletions appear to frequently induce loss of viability in Arabidopsis and thus are underrepresented in a mutant population [39]. Furthermore, a mutational dose that may be considered optimal may change from one species to another. Therefore, direct study of the effect of HII on wheat deletion size would be most desirable for this purpose.

In addition to presenting difficulties associated with deletion stacking in wheat, as suggested by this study, large deletions such as those present in the mutants assessed here may complicate reverse genetic approaches, since numerous other genes are removed with a gene of interest. Nevertheless, the resource used for this study may be powerful for certain research approaches. For example, it may be effective for forward genetic analyses, as large deletions contributing to a characteristic of interest should be relatively easy to identify by genomic analysis, particularly using modern high-throughput sequencing approaches [20]. Furthermore, the resource may be very useful to assist with functional validation of QTL via phenotypic characterisation of mutants featuring deletions of the QTL region.

Additionally, despite the redundancy of the wheat genome, in some cases deletions of all homoeoloci may not be required to obtain phenotypic alteration; the degree of functional redundancy amongst homoeologues seems likely to be both gene and homoeologue dependent. For example, Shaw *et al*. [40] have recently shown that loss of function of the B homoeologue of the *Photoperiod-1* (*Ppd-1*) gene confers substantially increased flowering time, while loss of function of the A homoeologue confers a more subtle change in flowering phenotype, and loss of function of the D homoeologue does not produce a phenotype independently (although effects are detected when combined with deletions of the B homoeologue). Whether a similar phenomenon can be observed for the putative susceptibility genes studied here is now under investigation through pathogen inoculation and defence gene expression analyses.

## Material and Methods

### Mutant population

The mutant population described in [15] was used for this study. To develop this population, heavy ion irradiation of grain of cv. Chara (AWB Seeds Ltd., Australia) was performed by the RIKEN-Japan RI-beam facility using a Neon ion beam at 50 Gy with LET at 63 KeV/mm^2^. An M2 population comprising approximately 9100 lines was produced with leaf material sampled for DNA extraction, and M3 seed indexed to M2 DNA samples.

### Routine plant growth

Plants were grown in either 48-cell trays (Kwikpot K48, Garden City Plastics, Australia) using UC mix with a sprinkling of Basacote Mini 3-month (Compo GmbH and Co), or 140 mm ANOVA pots (http://www.anovapot.com/index.php) using Searles Peat80 potting mix (JC & AT Searle PTY LTD, Australia).

### DNA extraction

Approximately 0.05 grams fresh leaf tissue was harvested into 96-tube racks (MTS-11–8-C-R; Fisher Biotec, Australia) and lyophilized. DNA extraction was performed by the Australian Genome Research Facility (Glen Osmond, Adelaide, Australia) using NucleoSpin Plant II kits (Macherey-Nagel) with on-column RNase digestion. DNA concentration was assessed using a Nanodrop 2000c (Thermo Scientific) and stored in indexed 96-well half skirt PCR plates (PCR-96M2-HS; Axygen, USA).

### Cloning and sequencing of *TaWRKY11* homoeologue fragments

A TBLASTN [41] search of the TIGR Rice Genome Annotation Database (http://blast.jcvi.org/euk-blast/index.cgi?project=osa1) using the protein sequence of Arabidopsis WRKY11 (AT4G31550.1) identified Os04g21950 as the best match (58.73% global similarity as assessed using ‘SIAS’ http://imed.med.ucm.es/Tools/sias.html). Following this, a TBLASTN search of the GrainGenes database (http://wheat.pw.usda.gov/GG2/index.shtml) with Os04g21950 identified the *Triticum aestivum* mRNA sequence BT009449 as the best match (84.75% global similarity). A subsequent TBLASTN search of the TAIR database (http://arabidopsis.org/index.jsp) with translated BT009449 identified AT4G31550.1 (*WRKY11*) as the best match (55.42% global similarity), indicating BT009449 as a likely candidate wheat homologue of *WRKY11*, and this gene was designated *TaWRKY11*. BT009449 sequence was used to design primers for amplification of genomic *TaWRKY11* sequence, using Primer Premier 5.1 (Premier Biosoft International, Palo Alto, CA). An ~ 950 bp genomic fragment was amplified from DNA extracted from wheat cv. ‘Chara’ using Phusion High-Fidelity DNA polymerase (Finnzymes Oy, Keilaranta 16 A, 02150 Espoo, Finland) with a manufacturer-reported error rate of 4.4 × 10^–7^. Products were cloned using a TOPO TA cloning kit (Invitrogen, Carlsbad, CA, USA) and plasmid preparations were produced using a QIAprep Spin Miniprep kit (Qiagen GmbH) in conjunction with a QIAcube (Qiagen GmbH) apparatus. The Australian Genome Research Facility (AGRF) plasmid sequencing service (AGRF, Brisbane, QLD, Australia) was used to obtain cloned fragment sequence using forward and reverse M13 primers. Sequence was analysed using Sequencher Version 4.9 Software (Gene Codes Corporation, Ann Arbor, MI, USA).

45 fragments were sequenced and three unique, putatively homoeologous sequences were identified (S1 Fig.) and deposited in GenBank (accession numbers KP036496, KP036497, and KP036498).

### High-throughput deletion screening

For *TaPFT1* and *TaPLDß1* deletion genotyping was performed using the method outlined in [15] and the homoeologue-specific, multiplexed TaqMan assays targeting *TaPFT1* and *TaPLDß1* described therein. Briefly, for a wheat gene of interest uniquely fluorophore-labelled TaqMan SNP-detection probes are designed to specifically target each homoeologue. Multiplex qPCR is performed using these probes and a gene specific primer mix, such that efficient fluorescence from each probe is observed in the presence of its corresponding homoeologue. Where a homoeolocus is deleted, fluorescence from its corresponding probe is inefficient or absent. By observing the fluorescence amplification plots of each fluorophore for a given reaction, the presence or absence of a specific homoeolocus can be determined.

For *TaWRKY11*, a modified version of the method outlined in [15] was used. Homoeologue-specific TaqMan minor groove binding, non-fluorescent quencher (MGB-NFQ) probes and a gene specific primer mix was designed as for the assays outlined in [15] (S7 Fig.; Table A in S1 File). However, for this assay it was found that two probes, WRKY11prb1FAM and WRKY11prb3NED, did not multiplex successfully. A functional ‘dual duplex’ assay for *TaWRKY11* was produced; for this assay, each sample was screened using two reactions, one containing WRKY11prb1FAM and WRKY11prb2VIC, and the other containing WRKY11prb3NED and WRK11prb2VIC (e.g. S3 and S4 Figs.). For both duplex assays 11 µL reactions containing 1000 nM forward primer mix, 1000 nM reverse primer mix, 5 µL TaqMan Universal PCR master mix (Life Technologies, Inc) and 2 µL ~ 100 ng/µL DNA were used. For the ‘FAM/VIC’ duplex, 150 nM FAM and 50 nM VIC labelled probes were used. For the ‘NED/VIC’ duplex, 200 nM NED and 50 nM VIC labelled probes were used. Cycling conditions were 50°C for 2 mins, 95°C for 10 mins, followed by 45 cycles of 95°C for 15s, 55°C for 30s and 60°C for 30s. qPCR screening was performed using an Applied Biosystems 7900HT (Applied Biosystems, Foster City, CA) or a ViiA7 Real-Time PCR System (Life Technologies, Inc).

### Development of plant material

#### Attempts to produce lines lacking two of the three homoeoloci for candidate genes

For *TaWRKY11*, *TaPFT1* and *TaPLDß1*, independent ‘primary’ mutant lines, possessing homozygous deletions for each of the individual homoeoloci of the genes were identified (Table B in S1 File). For *TaPFT1* and *TaPLDß1*, identification of these primary deletion mutants was reported in [15]; for *TaWRKY11* see Results. For each gene, multiple combinations of mutants lacking different homoeoloci were artificially crossed to produce F1 seed (Table 1). F1 seed was then selfed to produce F2 progeny and individual F2 progeny were planted and genotyped as described above. For lines putatively possessing homozygous deletions of two of the three homoeoloci (‘tetras’), several F3 seed was grown to re-genotype for confirmation and to produce bulked F4 seed.

#### Attempts to produce lines lacking all homoeologous copies of candidate genes

For *TaPLDß1*, crosses were made between tetra lines lacking A and D homoeoloci (AD-tetras) and B and D homoeoloci (BD-tetras) to produce F1 seed (Table 2). F1 seed was selfed to produce F2 progeny, and F2 progeny were planted and genotyped as described above.

For *TaWRKY11*, crosses between multiple independent tetra lines lacking the A and B homoeoloci (AB-tetras) and the D-homoeolocus primary mutant *tawrky11–300-d* were conducted (Table 2). F1 seed was selfed to produce F2 progeny and F2 progeny were planted and genotyped as described above.

### Amplicon sequencing to assess integrity of genes flanking *TaPFT1* by synteny to *Brachypodium distachyon*

A *Brachypodium distachyon* gene (Bradi4g27747) with 88.4% DNA sequence identity to the full length *TaPFT1* cDNA sequence (UniGene Ta.39294) was identified on Brachypodium chromosome 4 by a BLASTN search of the Phytozome database (http://www.phytozome.net/). This gene was designated *BdPFT1*. The Brachypodium genome annotation (version 1.92; http://www.phytozome.net/) was used to identify genes flanking *BdPFT1* at a range of distances (Material and Methods). Genes flanking *BdPFT1* at regular intervals (Table 3) within the ~4 Mb region from Bd4:30993677 to Bd4:35045079 were identified using the phytozome *Brachypodium distachyon* genome browser (http://www.phytozome.net/cgi-bin/gbrowse/brachy/). ‘Up’ is used to indicate the direction towards distal end of the short arm and ‘Down’ is used to indicate the opposite direction. Genes were selected based on the presence of intron/exon structure that would be likely to facilitate the design of primers to generate amplicons of ~ 500–1500 base pairs in length, with the primers anchored in exons and the amplicon spanning an intron/introns. BLASTN searches of wheat sequences within the NCBI (http://blast.ncbi.nlm.nih.gov/) and JCVI (http://blast.jcvi.org) databases were used to identify homologous wheat sequences to the Brachypodium genes selected (Table 3). Putative intron/exon structure of the wheat genes corresponding to the ESTs identified was visualized by aligning wheat EST to Brachypodium full gene sequence using the Spidey mRNA to genomics alignment tool (http://www.ncbi.nlm.nih.gov/spidey/). Primers were then designed as described above using Primer Premier 5.0 (Premier Biosoft International, Palo Alto, California). For each gene, two independent primer pairs (Table H in S1 File) were designed to increase the likelihood of obtaining at least one successful primer pair per gene. Primers were synthesized by Integrated DNA technologies (1710 Commercial Park Coralville, Iowa 52241, USA).

From each sample being analysed, PCR amplification was performed using all designed primer pairs in single-plex reactions. Individual primer pairs at 10 µM per primer eluted in DNase-free water were arranged in individual wells of a half-skirt 96-well PCR plate (PCR-96M2-HS; Axygen, USA). PCR master mix stock composed of 5 µL High Fidelity Phusion PCR Buffer (New England BioLabs, Inc.), 0.5 µL 10 mM dNTPs, 0.75 µL DMSO, 0.25 µL Phusion proofreading DNA polymerase and 15.5 µL DNase-free water per 22 µL was prepared in a 2.0 mL safe-lock PCR tube.

An Eppendorf EpMotion 5070 (Eppendorf AG, Hamburg, Germany) was used to combine 1.5 µL of a specific primer pair, 1.5 µL of 100 µM DNA and 22 µL PCR master mix into individual wells of a new half-skirt 96-well PCR plates (PCR-96M2-HS; Axygen, USA). PCR was performed on an Eppendorf Mastercycler ep gradient S (Eppendorf AG, Hamburg, Germany) using a touchdown protocol as follows: hot start at 98°C; 1 min at 98°C; 15 cycles with-1°C annealing temperature per cycle starting with 98°C for 15s, 70°C for 15s, 72°C for 30s; 25 cycles of 98°C for 15s, 58°C for 15s, 72°C for 30s; 72°C for 5 mins; hold at 22°C. PCR products from were visualized on a 1.5% agarose gel run at 90V for 65 mins against a 1 Kb Plus ladder (Life Technologies, Inc.) (e.g. S8).

For each individual sample, 10 µL of each successfully amplified PCR product was added to a single 2.0 mL PCR tube using the EpMotion 5070. The pooled amplicons were then purified using a QIAquick PCR purification kit (Qiagen, GmbH), visualized on 1.5% agarose and normalized to a concentration of ~ 100 ng/uL as assessed using a NanoDrop 2000 (Thermo Scientific, Inc.) using DNase free water. Amplicon sequencing was then performed by the Australian Genome Research Facility (AGRF; Brisbane, Australia). For each sample, pooled amplicons were enzymatically sheared using IonShear (Life Technologies, Inc.), purified with AmPure XP (Beckman Coulter, Inc.) and barcoded adapters for sequencing ligated following a standard Ion Torrent protocol. Samples were then pooled and sequenced across two Ion 318 chips (Life Technologies, Inc.) using an Ion Torrent PGM sequencer (Life Technologies, Inc.). Samples included on the first chip were: wild type Chara, wild type Chinese Spring, *tapft1–616-a*, *tapft1–157-b*, *tapft1–723-d*, and the ditellosomic control lines CS5AL-14. Samples included on the second chip were wild type Chara, wild type Chinese Spring, *tapft1–616-a*, *tapft1–85-a*, *tapft1–734-d*, *tapft1–757-d*, and the nullisomic-tetrasomic control lines ‘N5AT5B’ (lacking the 5A homoeologous chromosome), ‘N5BT5D’ (lacking 5B) or ‘N5DT5A’ (lacking 5D). The first chip produced 299.6 Mb of Q20 or better data; the second chip produced 795.4 Mb of Q20 or better data. The large discrepancy between data produced from the first and second chips was primarily attributed to a bubble formed on the first chip during loading; however, based on the proportion of Q20 bases from the first and second chips (62% and 84%, respectively) the overall run quality was higher for the second chip also.

Sample-specific sequences obtained were quality trimmed using CLC Genomics Workbench Version 5.1 (Qiagen, GmbH) using default settings for Ion Torrent data. A reference alignment approach was used to map reads to individual genes. For reference sequence, *A*. *tauschii* (D-genome progenitor of cultivated wheat) scaffolds containing sequence homologous to target amplicons was obtained by a BLASTN search of the whole *A*. *tauschii* genome sequence. For individual samples, reads were aligned to all reference sequences using criteria of minimum identity of 85%. SNP detection within all alignments was then performed using the CLC Genomics Workbench SNP detection tool using criteria of minimum allele frequency of 5% and minimum read coverage of 50x. Alignments were visually inspected to assess the extent of PCR duplication artefact with no substantial duplication observed.

SNP tables indexing SNPs detected within all reference alignments for individual samples were exported from CLC Genomics Workbench in CSV format. Data was formatted using custom-written Perl scripts (chara_cs_overlap.pl and pft1_flank.pl). To call genes as intact or deleted, the approach used was as follows. 1. For each gene, SNPs present in all wild type Chara E86 and Chinese Spring samples were considered useful; other SNPs were discarded as either accession-specific or unreliable. 2. Of these SNPs, those absent from the nullisomic-tetrasomic control lines ‘N5AT5B’ (lacking the 5A homoeologous chromosome), ‘N5BT5D’ (lacking 5B) or ‘N5DT5A’ (lacking 5D) were considered homoeologue-specific (S4 File). The presence of these homoeologue-specific SNPs was then assessed in *TaPFT1* primary mutant samples, lacking individual *TaPFT1* homoeologues. Where SNPs were consistently deleted from a mutant sample and its respective nullisomic-tetrasomic control, the gene was considered deleted in that mutant (S5 File). For four of the genes targeted, deleted SNPs were not identified in any of the nullisomic-tetrasomic controls. For the remaining 15 genes, intactness in the mutant and control samples is outlined in Table F in S1 File. In addition to the wild type and nullisomic-tetrasomic control accessions, the ditelosomic line CS5AL-14, from which *TaPFT1*-*A* was previously found to be absent [15] was included as a further control; A-homoeologue copies of the targeted genes were found to be consistently absent in this line and the nullisomic-tetrasomic control. Furthermore, one mutant sample, *tapft1–616-a*, was included in duplicate (once in each of the two Ion Torrent sequencing chips used). Identical deletion patterns were identified for both duplicates (Table F in S1 File).

### Comparison of physical size and gene content between Brachypodium and wheat in the region of PFT1

To compare the physical size and overall gene content in wheat and Brachypodium in the regions harbouring *BdPFT1* and *TaPFT1*, we undertook a strategy using the CSS in conjunction with the genome sequences of *Triticum urartu* [42] and *A*. *tauschii* [43] (‘A’ and ‘D’ genome diploid progenitors of hexaploid wheat, respectively), as follows.

cDNA sequence for all Brachypodium genes in the region flanking *BdPFT1* from Bd4:30993677 to Bd4:35045079, targeted for the approach outlined above, was obtained from phytozome using the ‘BioMart’ tool (http://www.phytozome.net/biomart/martview/6d4d3f098f4a29a be2240361a1c1ded6). A BLASTN search of gene sequences extracted from the CSS using the associated GTF file (http://plants.ensembl.org/info/website /ftp/index.html) in conjunction with a custom Perl script (gtf_to_genes.pl) was performed, and the best match for all genes was identified. This sequence was then used for a MEGABLAST search against the entire CSS, and additional matches with an e-value of zero (highly likely to be homoeologues/gene duplications) were identified.

Data was formatted using custom Python scripts (PFT1_flank_CSS_hits_step1.py, PFT1_flank_CSS_hits_step2.py, and PFT1_flank_CSS_hits_step3.py) to assess whether homologues of these Brachypodium genes flanking *BdPFT1* were present on the long arms of the group 5 chromosome series, previously determined to harbour *TaPFT1* [15]. Taking into account the high degree of gene duplication within the wheat genome [44] and the incomplete nature of the CSS, for each gene we performed an assessment of the conservation of synteny between Brachypodium and wheat as follows.

Where no matches were identified on any of the group 5 chromosome long arms, this was taken as evidence of a translocation of the gene in wheat relative to Brachypodium. Where a match was identified on a given group 5 chromosome long arm, and no match of equal or greater identity and length was identified outside of the group 5 long arms, it was considered likely that this match represented a homoeologue. Where a match was identified on a given group 5 long arm, but a match of equal or greater length and identity was identified outside of the chromosome 5 long arms, it was considered uncertain whether this match represented a homoeologue or a gene duplication located within the syntenic chromosomal region. If a likely homoeologue was identified on at least one of the group 5 long arms, this was taken as evidence of conservation of synteny for that gene between wheat and Brachypodium. Alternatively, if all matches on the chromosome 5 long arms were considered uncertain by the criteria above, it was considered unclear whether synteny was conserved between Brachypodium and wheat for that gene. Based on these criteria, an overview of the conservation of synteny in this region is provided in S3 File. Furthermore, functional descriptions of Brachypodium genes within regions syntenic to deletion regions in wheat were assigned using BLAST2GO software [45] with standard BLAST, mapping and annotation parameters.

Data restricted to the genes analysed using the amplicon sequencing approach is presented in Table G in S1 File.

The size of the genomic regions harbouring genes for which synteny appeared conserved was estimated using the draft genome sequences of both *T*. *urartu* and *A*. *tauschii* which contain an estimated 74% and 73% of the genome in scaffolds longer than 10 kb, respectively [42], [43]. The length of unique scaffolds harbouring genes for which synteny appeared conserved in this region was calculated for *T*. *urartu* and *A*. *tauschii* using the Biopython (http://biopython.org/wiki/Biopython) SeqIO module.

### Statistical analysis

Chi-square contingency analysis was performed using GraphPad software (GraphPad Software Inc., 7825 Fay Avenue, Suite 230, La Jolla CA 92037).

## Supporting Information

S1 Fig *TaWRKY11* sequence alignments. 1A. Alignment of putatively homoeologous *TaWRKY11* sequence fragments obtained from wheat cv. Chara. 1B. Global identity amongst *TaWRKY11* fragments.
(TIF)

S2 Fig Fluorescence profiles of group 1 nullisomic-tetrasomic accessions using the high-throughput *TaPLDß1* assay. Fluorescence profiles using the *TaPLDß1* high-throughput assay from wild type Chinese Spring (all homoeologues intact) and the nullisomic-tetrasomic accessions N1AT1B (1A chromosomes absent; four 1B chromosomes); N1BT1A (1B chromosomes absent; four 1A chromosomes); N1DT1B (1D chromosomes absent; four 1B chromosomes). Inefficient/absent fluorescence from a probe for a given nullisomic-tetrasomic accession indicates that the probe targets the homoeologous copy of the gene located on the absent homoeologous chromosome. All images incorporate duplicate reactions and duplicate signals from each probe.
(TIF)

S3 Fig *TaWRKY11* screening of nullisomic-tetrasomic accessions with ‘dual duplex’ high-throughput assay. Fluorescence profiles from wild type Chinese Spring (all homoeologues intact) and the nullisomic-tetrasomic accessions N2BT2A (2B chromosomes absent; four 1A chromosomes) and N2BT2A (2D chromosomes absent; four 1A chromosomes). Screening was performed in ‘dual duplex’ assays (see Materials and Methods). 3A. Profiles for the WRKY11prbFAM/WRK11prbVIC duplex. 3B. Profiles for the WRKY11prbNED/WRKY11prbVIC duplex. Inefficient/absent fluorescence from a probe for a given nullisomic-tetrasomic accession indicates that the probe targets the homoeologous copy of the gene located on the absent homoeologous chromosome. All images incorporate duplicate reactions and duplicate signals from each probe.
(TIF)

S4 Fig Fluorescence profiles of *TaWRKY11* tetras detected using the ‘dual duplex’ high-throughput screening method. 5A. Profiles for the WRKY11prbFAM/WRK11prbVIC duplex. 5B. Profiles for the WRKY11prbNED/WRKY11prbVIC duplex. All images incorporate duplicate reactions and duplicate signals from each probe.
(TIF)

S5 Fig Fluorescence profiles of *TaPLDß1* tetras detected using the high-throughput screening method. Note that for A-B tetras some off-target fluorescence from the A-specific probe was routinely observed. However the signal could be clearly distinguished from that of the intact A target. All images incorporate duplicate reactions and duplicate.
(TIF)

S6 Fig Male sterility in *TaPLDß1* hexa lines. 4A. A *TaPLDß1* hexa line with spikelets dissected demonstrating unemerged, sterile anthers. 4B. A *TaPLDß1* hexa fertilized with wild type pollen, at a similar stage of development as that depicted in 4A. 4C. Primary spikes from wild type Chara and six *TaPLDß1* hexa lines at grain fill stage. 4D. Wild type Chara and three hexas spikes at maturity.
(TIF)

S7 Fig Design of primers and probes for high-throughput deletion screening of *TaWRKY11*. Light blue highlight represents binding sites for forward primers. Yellow highlight indicates binding sites for reverse primers. Purple, green and dark blue highlights indicate binding sites for D, B and A-specific TaqMan probes, respectively. Red highlight indicates polymorphism between homoeologous sequences.
(TIF)

S8 Fig Amplicons from genes putatively flanking *TaPFT1* from wild type Chara and Chinese Spring control samples. The bands from two independent primer pairs for each target are shown (e.g. U50K = products from primer pairs targeting the wheat homoeologue of the Brachypodium gene 50 Kb up from *BdPFT1*; Materials and Methods). Failed primers (one each for D100K and U1M) were excluded from sequencing. POS is a positive control primer pair.
(TIF)

S9 Fig Binding sites for *TaWRKY11* high-throughput assay within CSS sequences. Dark blue indicates probe binding site (probe binds to the reverse strand), light blue and yellow indicate primer sequences for the high-throughput assay (Table A in S1 File). 9A. WRKY11prbVIC binding sites within *TaWRKY11* sequence identified within the CSS sequence on chromosome 2AL. 9B. WRKY11prbNED binding sites within *TaWRKY11* sequence identified within the CSS sequence on chromosome 2BL. Dark blue indicates probe binding site (probe binds to the reverse strand), light blue and yellow indicate primer sequences for the high-throughput assay.
(TIF)

S10 Fig Gel based validation of *TaPLDß1* hexas and *TaWRKY11* and *TaPFT1* tetras. **10A.** Products of a *TaPFT1* CAPS assay [15] from the wild type control (WT), A, B and D primaries (A1, B1 and D1) and AD and BD tetras (AD1, AD2, BD1 and BD2). 10B. Products of a *TaWRKY11* homoeologue-specific CAPS assay (Materials and Methods). A, B and D specific bands are indicated with yellow, red and orange arrows, respectively. The presence of all bands is observed in the wild type control (WT). A, B and D primary mutants (A1, B1 and D1) and AB and AD-tetras (AB1, AB2, AD1 and AD2) lack the respective homoeologue-specific fragments. 10C. PCR products from a duplex reaction with a universal *TaPLDß1* primer mix (targeting all homoeologues) and control primers. Amplification of the control band but not the *TaPLDß1* band in lines H1—H8 indicate the absence of all *TaPLDß1* gene copies in these lines. Amplification of the control band and the *TaPLDß1* band from one putative hexa (labelled ‘X’) and the wild type (WT) indicates the presence of at least one copy of *TaPLDß1* in these lines.
(TIF)

S1 File Table A, Details of the application of the high throughput screening method to identify *TaWRKY11* deletions. **Table B, *TaWRKY11*, *TaPFT1* and *TaPLDß1* primary mutant accessions used for this study. Table C, Chromosomal location of candidate genes and homoeologous copy targeted by individual fluoro-labelled probes. Table D, Summary of tetras selected for further development and assessment.** All listed accessions had genotype confirmed at the F3 stage. **Table E, Expected genotypic ratios as detected by the probe screen of F2 progeny used in this study.** Upper case indicates intact homoeologue and lower case indicates deleted homoeologue. Index is provided for reference to Tables 1 and 2. E.g. Index 1 refers to the expected detected genotypic ratio within an F2 progeny from a cross between an A-deletion primary mutant and a B-deletion primary mutant. **Table F, Intactness of wheat homologues of genes flanking *BdPFT1* in control and mutant lines.** A green cell containing a ✓indicates that the gene is intact. A red cell containing an X indicates that the gene is deleted. A white cell containing a? indicates that homoeologue-specific SNPs could not be identified for that homoeologue and intactness is unknown. **Table G, Assessment of conservation of synteny of genes flanking *PFT1*, targeted for the amplicon sequencing approach to assess deletion size in wheat.** Under ‘5AL homoeologue’, ‘5BL homoeologue’, and ‘5DL homoeologue’, Yes/No indicates whether or not a homoeologue was identified within the CSS on that chromosome arm by *in silico* analysis; ‘?’ indicates a lack of confidence. Under ‘Translocated *in vitro*’, Yes/No indicates whether or not a translocation is indicated by the amplicon sequencing approach. Under ‘Translocated *in silico*’, Yes/No indicates whether, overall, a translocation is indicated by *in silico* analysis; ‘?’ indicates a lack of confidence. **Table H, Primers used for amplicon sequencing approach to assess the size of deletions removing *TaPFT1*. Table I, MEGABLAST matches (E-value = 0) of candidate gene sequence obtained from wheat cv. Chara against Chinese Spring ‘Chromosomal Survey Sequence’.**
(DOCX)

S2 File Supplementary Material and Methods, and Supplementary Results. (DOCX)

S3 File Synteny in the region 2 Mb ‘Up’ to 2 Mb ‘Down’ of *PFT1* in Brachypodium and wheat. Based on comparison of the *Brachypodium distachyon* 1.92 annotation (http://www.phytozome.net/) and the wheat Chromosomal Survey Sequences (http://plants.ensembl.org/index.html) as described in Materials and Methods. For each Brachypodium gene the presence of the putative orthologue across the wheat chromosome 5 series long arms is indicated. On the basis of this information, likely translocations are indicated. Homologues of genes with descriptions labelled ‘A’ and ‘B’, and ‘C’ are required for viability in Arabidopsis; grey shading region indicates the minimum size of the region that was incompatible for deletion stacking, see Fig. 2 and Discussion.
(XLSX)

S4 File Identification of homoeologous chromosome-specific SNPs within amplicons from *TaPFT1* flanking genes. As described in Materials and Methods. ‘WT_1’ and ‘WT_2’ are duplicate Chara samples, and ‘CS_1’ and ‘CS_2’ are duplicate Chinese Spring samples, one from each Ion Torrent chip; ‘N5A’ is the nullisomic-tetrasomic Chinese Spring accession N5AT5B, ‘N5D’ is N5DT5A, and ‘N5B’ is N5BT5D; ‘scaffold_ID’ is the ID of the *Aegilops tauchii* genome scaffold used for reference alignment; ‘gene_ID’ is the accession given with respect to location from *TaPFT1*; ‘ref_position’ is the position of the SNP with respect to the reference sequence; ‘ref_type’ is the reference base at the SNP position; ‘num_variants’ is the number of SNP variants at that position; ‘allele_variants’ is SNP variant detected in at least one sample at a specific reference position; frequency indicates the percentage of bases for each variant; coverage is the sequencing coverage for a specific SNP position.
(XLSX)

S5 File Assessment of the intactness of homoeologous chromosome-specific SNPs within amplicons from *TaPFT1* flanking genes in *TaPFT1* mutant samples. As described in Materials and Methods. ‘WT_1’ and ‘WT_2’ are duplicate Chara samples, and ‘CS_1’ and ‘CS_2’ are duplicate Chinese Spring samples, one from each Ion Torrent chip; ‘N5A’ is the nullisomic-tetrasomic Chinese Spring accession N5AT5B, ‘N5D’ is N5DT5A, and ‘N5B’ is N5BT5D; ‘616-A_1’ and ‘616-A_2’ are duplicate *tapft1–616-a* samples, one from each Ion Torrent chip; ‘85-A’ is *tapft1–85-a*; ‘5AL-14’ is the ditelosomic Chinese Spring accession CS5AL-14, ‘723-D’ is *tapft1–723-d*; ‘757-D’ is *tapft1–757-d*; ‘734-D’ is *tapft1–734-d*; ‘157-B’ is *tapft1–157-b*; ‘scaffold_ID’ is the ID of the *Aegilops tauchii* genome scaffold used for reference alignment; ‘gene_ID’ is the accession given with respect to location from *TaPFT1*; ‘ref_position’ is the position of the SNP with respect to the reference sequence; ‘ref_type’ is the reference base at the SNP position; ‘num_variants’ is the number of SNP variants at that position; ‘allele_variants’ is SNP variant detected in at least one sample at a specific reference position; frequency indicates the percentage of bases for each variant; coverage is the sequencing coverage for a specific SNP position.
(XLSX)

S6 File Assessment of translocation of genes from the region from ~ 1Mb to ~ 2Mb ‘Up’ from *BdPFT1* to regions within the wheat chromosome 4 series. Based on comparison of the *Brachypodium distachyon* 1.92 annotation (http://www.phytozome.net/) and the wheat Chromosomal Survey Sequences (http://plants.ensembl.org/index.html) as described in Materials and Methods. For each Brachypodium gene the presence of the putative orthologue within the 4AL, 4BS, and 4DS wheat chromosomal regions is indicated.
(XLSX)
