# Supplementary figures and images for: An Assessment of Heavy Ion Irradiation Mutagenesis for Reverse Genetics in Wheat (Triticum aestivum L.)
Source: PLoS One. 2015 Feb 26;10(2):e0117369. doi: 10.1371/journal.pone.0117369 (PMC4342231; doi:10.1371/journal.pone.0117369)

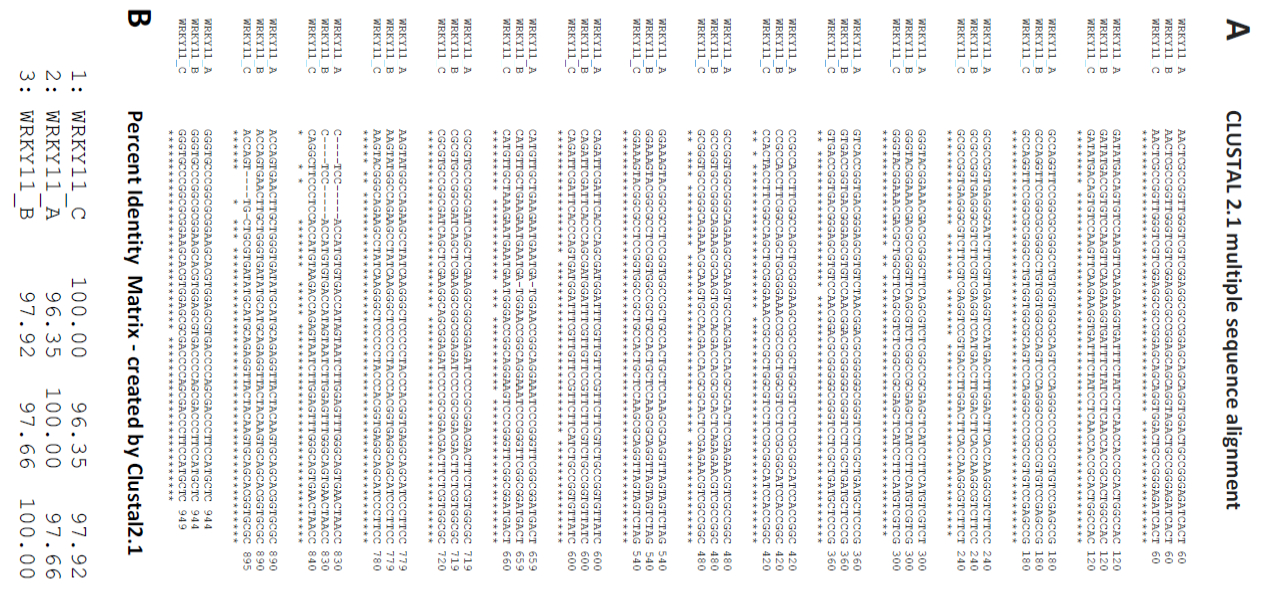

Supplement: S1 Fig — 1A. Alignment of putatively homoeologous TaWRKY11 sequence fragments obtained from wheat cv. Chara. 1B. Global identity amongst TaWRKY11 fragments. (TIF) [file pone.0117369.s001.tif]

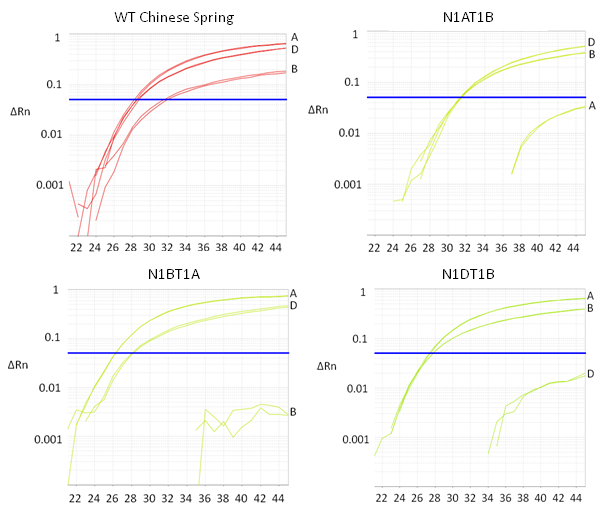

Supplement: S2 Fig — Fluorescence profiles using the TaPLDß1 high-throughput assay from wild type Chinese Spring (all homoeologues intact) and the nullisomic-tetrasomic accessions N1AT1B (1A chromosomes absent; four 1B chromosomes); N1BT1A (1B chromosomes absent; four 1A chromosomes); N1DT1B (1D chromosomes absent; four 1B chromosomes). Inefficient/absent fluorescence from a probe for a given nullisomic-tetrasomic accession indicates that the probe targets the homoeologous copy of the gene located on the absent homoeologous chromosome. All images incorporate duplicate reactions and duplicate signals from each probe. (TIF) [file pone.0117369.s002.tif]

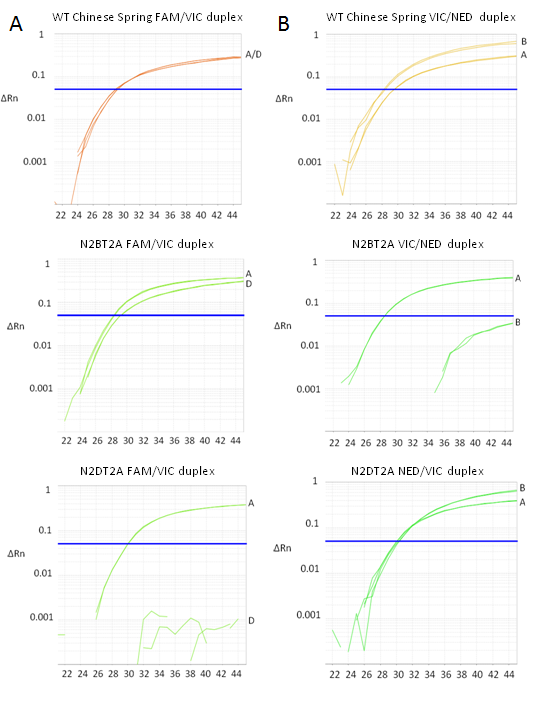

Supplement: S3 Fig — Fluorescence profiles from wild type Chinese Spring (all homoeologues intact) and the nullisomic-tetrasomic accessions N2BT2A (2B chromosomes absent; four 1A chromosomes) and N2BT2A (2D chromosomes absent; four 1A chromosomes). Screening was performed in ‘dual duplex’ assays (see Materials and Methods). 3A. Profiles for the WRKY11prbFAM/WRK11prbVIC duplex. 3B. Profiles for the WRKY11prbNED/WRKY11prbVIC duplex. Inefficient/absent fluorescence from a probe for a given nullisomic-tetrasomic accession indicates that the probe targets the homoeologous copy of the gene located on the absent homoeologous chromosome. All images incorporate duplicate reactions and duplicate signals from each probe. (TIF) [file pone.0117369.s003.tif]

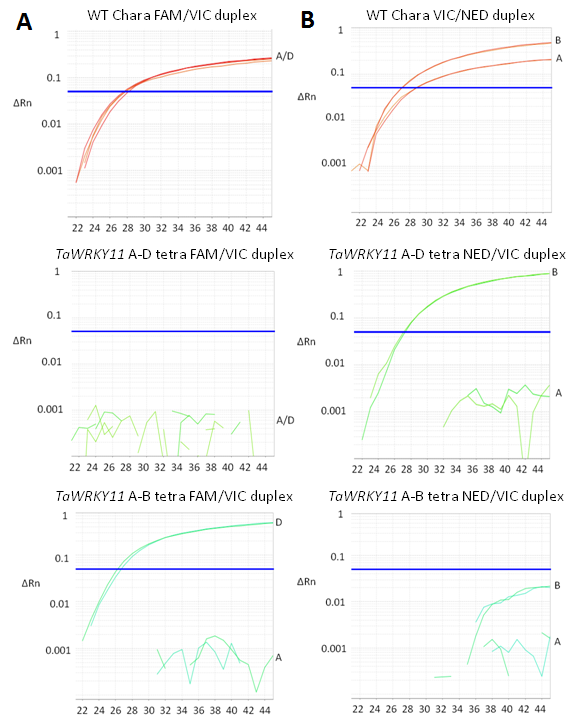

Supplement: S4 Fig — 5A. Profiles for the WRKY11prbFAM/WRK11prbVIC duplex. 5B. Profiles for the WRKY11prbNED/WRKY11prbVIC duplex. All images incorporate duplicate reactions and duplicate signals from each probe. (TIF) [file pone.0117369.s004.tif]

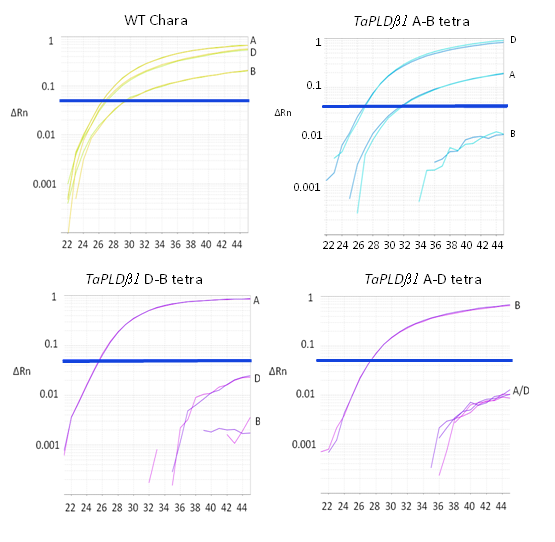

Supplement: S5 Fig — Note that for A-B tetras some off-target fluorescence from the A-specific probe was routinely observed. However the signal could be clearly distinguished from that of the intact A target. All images incorporate duplicate reactions and duplicate. (TIF) [file pone.0117369.s005.tif]

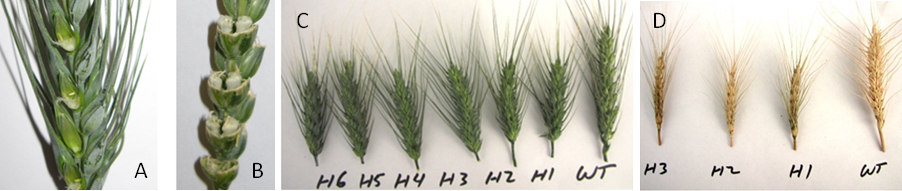

Supplement: S6 Fig — 4A. A TaPLDß1 hexa line with spikelets dissected demonstrating unemerged, sterile anthers. 4B. A TaPLDß1 hexa fertilized with wild type pollen, at a similar stage of development as that depicted in 4A. 4C. Primary spikes from wild type Chara and six TaPLDß1 hexa lines at grain fill stage. 4D. Wild type Chara and three hexas spikes at maturity. (TIF) [file pone.0117369.s006.tif]

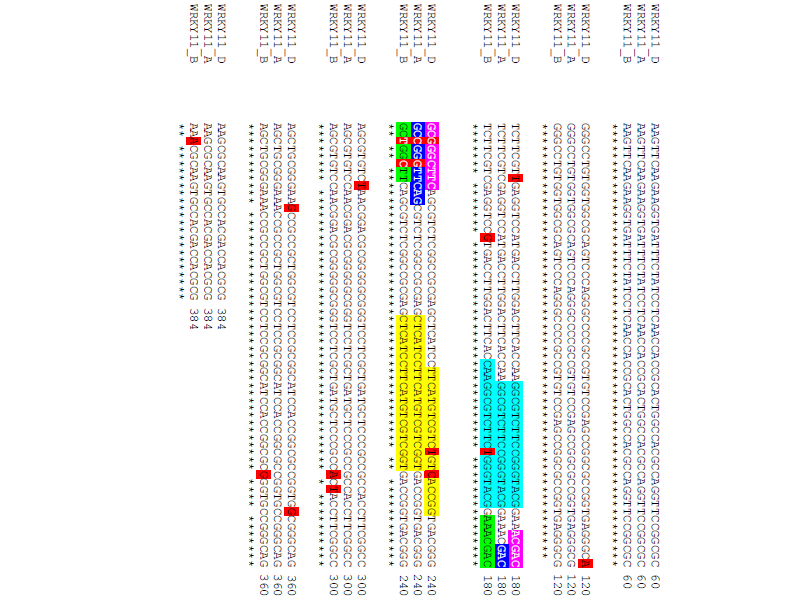

Supplement: S7 Fig — Light blue highlight represents binding sites for forward primers. Yellow highlight indicates binding sites for reverse primers. Purple, green and dark blue highlights indicate binding sites for D, B and A-specific TaqMan probes, respectively. Red highlight indicates polymorphism between homoeologous sequences. (TIF) [file pone.0117369.s007.tif]

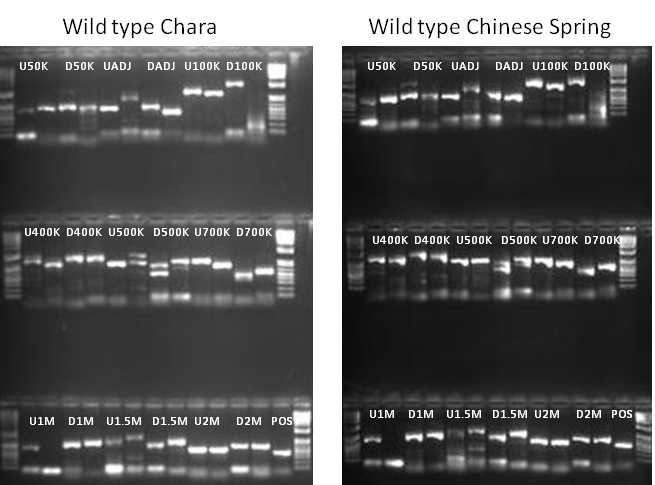

Supplement: S8 Fig — The bands from two independent primer pairs for each target are shown (e.g. U50K = products from primer pairs targeting the wheat homoeologue of the Brachypodium gene 50 Kb up from BdPFT1; Materials and Methods). Failed primers (one each for D100K and U1M) were excluded from sequencing. POS is a positive control primer pair. (TIF) [file pone.0117369.s008.tif]

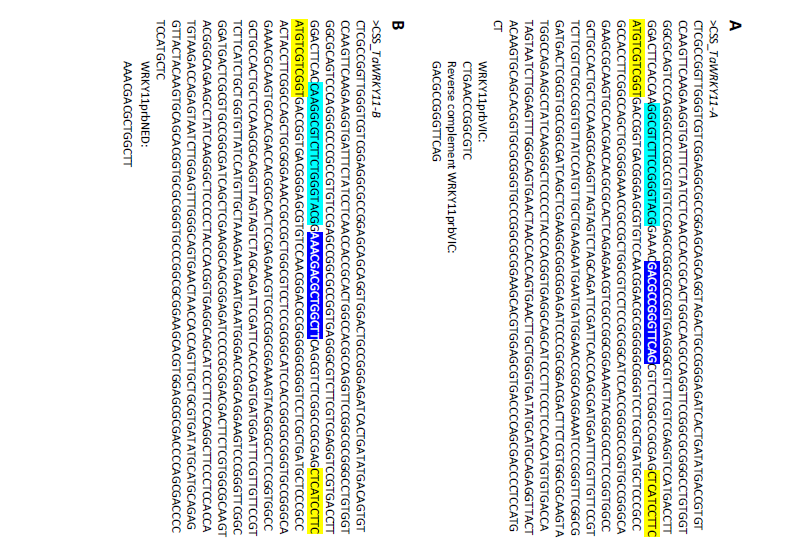

Supplement: S9 Fig — Dark blue indicates probe binding site (probe binds to the reverse strand), light blue and yellow indicate primer sequences for the high-throughput assay (Table A in S1 File). 9A. WRKY11prbVIC binding sites within TaWRKY11 sequence identified within the CSS sequence on chromosome 2AL. 9B. WRKY11prbNED binding sites within TaWRKY11 sequence identified within the CSS sequence on chromosome 2BL. Dark blue indicates probe binding site (probe binds to the reverse strand), light blue and yellow indicate primer sequences for the high-throughput assay. (TIF) [file pone.0117369.s009.tif]

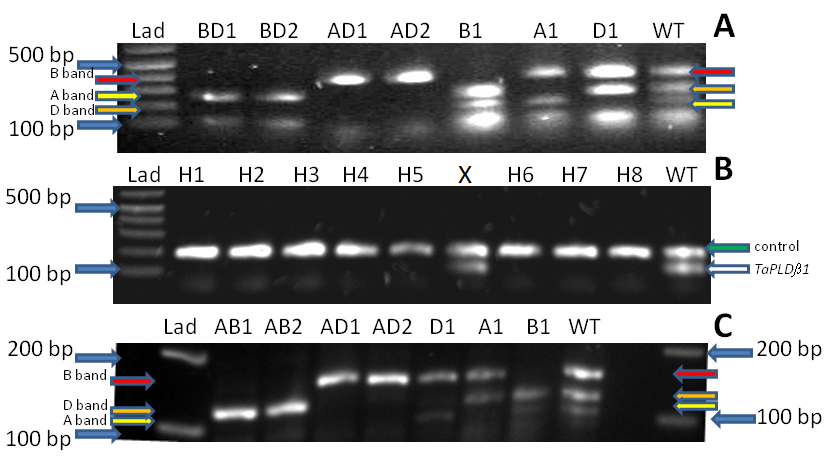

Supplement: S10 Fig — 10A. Products of a TaPFT1 CAPS assay [15] from the wild type control (WT), A, B and D primaries (A1, B1 and D1) and AD and BD tetras (AD1, AD2, BD1 and BD2). 10B. Products of a TaWRKY11 homoeologue-specific CAPS assay (Materials and Methods). A, B and D specific bands are indicated with yellow, red and orange arrows, respectively. The presence of all bands is observed in the wild type control (WT). A, B and D primary mutants (A1, B1 and D1) and AB and AD-tetras (AB1, AB2, AD1 and AD2) lack the respective homoeologue-specific fragments. 10C. PCR products from a duplex reaction with a universal TaPLDß1 primer mix (targeting all homoeologues) and control primers. Amplification of the control band but not the TaPLDß1 band in lines H1—H8 indicate the absence of all TaPLDß1 gene copies in these lines. Amplification of the control band and the TaPLDß1 band from one putative hexa (labelled ‘X’) and the wild type (WT) indicates the presence of at least one copy of TaPLDß1 in these lines. (TIF) [file pone.0117369.s010.tif]
