# Supplementary material for: An Assessment of Heavy Ion Irradiation Mutagenesis for Reverse Genetics in Wheat (Triticum aestivum L.)
Source: PLoS One. 2015 Feb 26;10(2):e0117369. doi: 10.1371/journal.pone.0117369 (PMC4342231; doi:10.1371/journal.pone.0117369)
Supplement: S1 File — Table B, TaWRKY11, TaPFT1 and TaPLDß1 primary mutant accessions used for this study. Table C, Chromosomal location of candidate genes and homoeologous copy targeted by individual fluoro-labelled probes. Table D, Summary of tetras selected for further development and assessment. All listed accessions had genotype confirmed at the F3 stage. Table E, Expected genotypic ratios as detected by the probe screen of F2 progeny used in this study. Upper case indicates intact homoeologue and lower case indicates deleted homoeologue. Index is provided for reference to Tables 1 and 2. E.g. Index 1 refers to the expected detected genotypic ratio within an F2 progeny from a cross between an A-deletion primary mutant and a B-deletion primary mutant. Table F, Intactness of wheat homologues of genes flanking BdPFT1 in control and mutant lines. A green cell containing a ✓indicates that the gene is intact. A red cell containing an X indicates that the gene is deleted. A white cell containing a? indicates that homoeologue-specific SNPs could not be identified for that homoeologue and intactness is unknown. Table G, Assessment of conservation of synteny of genes flanking PFT1, targeted for the amplicon sequencing approach to assess deletion size in wheat. Under ‘5AL homoeologue’, ‘5BL homoeologue’, and ‘5DL homoeologue’, Yes/No indicates whether or not a homoeologue was identified within the CSS on that chromosome arm by in silico analysis; ‘?’ indicates a lack of confidence. Under ‘Translocated in vitro’, Yes/No indicates whether or not a translocation is indicated by the amplicon sequencing approach. Under ‘Translocated in silico’, Yes/No indicates whether, overall, a translocation is indicated by in silico analysis; ‘?’ indicates a lack of confidence. Table H, Primers used for amplicon sequencing approach to assess the size of deletions removing TaPFT1. Table I, MEGABLAST matches (E-value = 0) of candidate gene sequence obtained from wheat cv. Chara against Chinese Spring ‘Chromosomal [file pone.0117369.s011.docx]

**Table A.** Details of the application of the high throughput screening method to identify *TaWRKY11* deletions.

| **Gene** | **Forward primers** | **Reverse primers** | **Homoeologue specific probes** | **Mutants screened** | **Deletions mutants confirmed** |
| --- | --- | --- | --- | --- | --- |
| *TaWRKY11* | FWRKY11gsprbA.B: GGCGTCTTCCGGGTACG FWRKY11gsprbC: CAAGGCGTCTTCTGGGTACG | RWRKY11gsprbB.C: ACCGACGACATGAAGGATGAG RWRKY11gsprbA: CCGGTGACAGACGACATGAA | WRKY11prbFAM: GAAGCCCGCGTCGT (binds to reverse strand) WRKY11prbVIC: CTGAACCCGGCGTC (binds to reverse stand) WRKY11prbNED: AAACGACGCTGGCTT | 2496 | 7 lines in total:  4 *TaWRKY11-A* deletions;  2 *TaWRKY11-B* deletions;  1 *TaWRKY11-D* deletion |

**Table B.** *TaWRKY11*, *TaPFT1* and *TaPLDß1* primary mutant accessions used for this study.

| **Gene deletion** | **Mutant accession** | **Homoeologue deleted** |
| --- | --- | --- |
| *TaWRKY11* | *tawrky11-178-a* | *TaWRKY11-A* |
| *TaWRKY11* | *tawrky11-227-a* | *TaWRKY11-A* |
| *TaWRKY11* | *tawrky11-286-a* | *TaWRKY11-A* |
| *TaWRKY11* | *tawrky11-417-a* | *TaWRKY11-A* |
| *TaWRKY11* | *tawrky11-87-b* | *TaWRKY11-B* |
| *TaWRKY11* | *tawrky11-743-b* | *TaWRKY11-B* |
| *TaWRKY11* | *tawrky11-300-d* | *TaWRKY11-D* |
| *TaPFT1* | *tapft1-85-a* | *TaPFT1-A* |
| *TaPFT1* | *tapft1-616-a* | *TaPFT1-A* |
| *TaPFT1* | *tapft1-157-b* | *TaPFT1-B* |
| *TaPFT1* | *tapft1-723-d* | *TaPFT1-D* |
| *TaPFT1* | *tapft1-734-d* | *TaPFT1-D* |
| *TaPFT1* | *tapft1-757-d* | *TaPFT1-D* |
| *TaPLDß1* | *tapldß1-208-a* | *TaPLDß1-A* |
| *TaPLDß1* | *tapldß1-334-a* | *TaPLDß1-A* |
| *TaPLDß1* | *tapldß1-690-a* | *TaPLDß1-A* |
| *TaPLDß1* | *tapldß1-150-b* | *TaPLDß1-B* |
| *TaPLDß1* | *tapldß1-855-b* | *TaPLDß1-B* |
| *TaPLDß1* | *tapldß1-147-d* | *TaPLDß1-D* |
| *TaPLDß1* | *tapldß1-934-d* | *TaPLDß1-D* |

**Table C.** Chromosomal location of candidate genes and homoeologous copy targeted by individual fluoro-labelled probes.

| **Gene name** | **Chromosome** | **A-homoeologue** | **B-homoeologue** | **D-homoeologue** |
| --- | --- | --- | --- | --- |
| *TaPFT1* | 5 | PFT1prbVIC | PFT1prbFAM | PFT1prbNED |
| *TaPLDß1* | 1 | PLDB1prbFAM | PLDB1prbVIC | PLDB1prbNED |
| *TaWRKY11* | 2 | WRKY11prbVIC | WRKY11prbNED | WRKY11prbFAM |

**Table D.** Summary of tetras selected for further development and assessment. All listed accessions had genotype confirmed at the F3 stage.

| **Gene deleted** | **Mutant accession** | **Male parent** | **Female parent** |
| --- | --- | --- | --- |
| *TaPFT1* | *tapft1-ad-1* | *tapft1-85-a* | *tapft1-734-d* |
| *TaPFT1* | *tapft1-ad-2* | *tapft1-85-a* | *tapft1-734-d* |
| *TaPFT1* | *tapft1-ad-3* | *tapft1-85-a* | *tapft1-734-d* |
| *TaPFT1* | *tapft1-ad-4* | *tapft1-85-a* | *tapft1-734-d* |
| *TaPFT1* | *tapft1-ad-5* | *tapft1-85-a* | *tapft1-734-d* |
| *TaPFT1* | *tapft1-bd-1* | *tapft1-157-b* | *tapft1-734-d* |
| *TaPFT1* | *tapft1-bd-2* | *tapft1-157-b* | *tapft1-734-d* |
| *TaPFT1* | *tapft1-bd-3* | *tapft1-157-b* | *tapft1-734-d* |
| *TaPFT1* | *tapft1-bd-4* | *tapft1-157-b* | *tapft1-734-d* |
| *TaPFT1* | *tapft1-bd-5* | *tapft1-157-b* | *tapft1-734-d* |
| *TaPFT1* | *tapft1-bd-7* | *tapft1-157-b* | *tapft1-757-d* |
| *TaPFT1* | *tapft1-bd-8* | *tapft1-157-b* | *tapft1-734-d* |
| *TaPFT1* | *tapft1-bd-9* | *tapft1-157-b* | *tapft1-734-d* |
| *TaPFT1* | *tapft1-bd-10* | *tapft1-157-b* | *tapft1-734-d* |
| *TaWRKY11* | *tawrky11-ab-1* | *tawrky11-87-b* | *tawrky11-286-a* |
| *TaWRKY11* | *tawrky11-ab-2* | *tawrky11-87-b* | *tawrky11-417-a* |
| *TaWRKY11* | *tawrky11-ab-3* | *tawrky11-87-b* | *tawrky11-417-a* |
| *TaWRKY11* | *tawrky11-ab-4* | *tawrky11-87-b* | *tawrky11-417-a* |
| *TaWRKY11* | *tawrky11-ab-5* | *tawrky11-743-b* | *tawrky11-286-a* |
| *TaWRKY11* | *tawrky11-ab-6* | *tawrky11-743-b* | *tawrky11-286-a* |
| *TaWRKY11* | *tawrky11-ab-7* | *tawrky11-743-b* | *tawrky11-286-a* |
| *TaWRKY11* | *tawrky11-ad-1* | *tawrky11-227-a* | *tawrky11-300-d* |
| *TaWRKY11* | *tawrky11-ad-3* | *tawrky11-417-a* | *tawrky11-300-d* |
| *TaWRKY11* | *tawrky11-ad-4* | *tawrky11-417-a* | *tawrky11-300-d* |
| *TaWRKY11* | *tawrky11-ad-5* | *tawrky11-417-a* | *tawrky11-300-d* |
| *TaWRKY11* | *tawrky11-ad-8* | *tawrky11-286-a* | *tawrky11-300-d* |
| *TaWRKY11* | *tawrky11-ad-9* | *tawrky11-286-a* | *tawrky11-300-d* |
| *TaWRKY11* | *tawrky11-ad-10* | *tawrky11-286-a* | *tawrky11-300-d* |
| *TaPLDß1* | *tapldb1-ad-1* | *tapldb1-147-d* | *tapldb1-334-a* |
| *TaPLDß1* | *tapldb1-ad-2* | *tapldb1-147-d* | *tapldb1-334-a* |
| *TaPLDß1* | *tapldb1-ad-3* | *tapldb1-147-d* | *tapldb1-334-a* |
| *TaPLDß1* | *tapldb1-ad-4* | *tapldb1-147-d* | *tapldb1-334-a* |
| *TaPLDß1* | *tapldb1-ad-5* | *tapldb1-781-a* | *tapldb1-934-d* |
| *TaPLDß1* | *tapldb1-ad-6* | *tapldb1-781-a* | *tapldb1-934-d* |
| *TaPLDß1* | *tapldb1-ad-7* | *tapldb1-781-a* | *tapldb1-934-d* |
| *TaPLDß1* | *tapldb1-bd-1* | *tapldb1-855-b* | *tapldb1-147-d* |
| *TaPLDß1* | *tapldb1-bd-2* | *tapldb1-855-b* | *tapldb1-147-d* |
| *TaPLDß1* | *tapldb1-bd-3* | *tapldb1-855-b* | *tapldb1-147-d* |
| *TaPLDß1* | *tapldb1-bd-4* | *tapldb1-934-d* | *tapldb1-150-b* |
| *TaPLDß1* | *tapldb1-bd-5* | *tapldb1-934-d* | *tapldb1-150-b* |
| *TaPLDß1* | *tapldb1-ad-8* | *tapldb1-147-d* | *tapldb1-334-a* |
| *TaPLDß1* | *tapldb1-ad-9* | *tapldb1-147-d* | *tapldb1-334-a* |
| *TaPLDß1* | *tapldb1-ad-10* | *tapldb1-147-d* | *tapldb1-334-a* |
| *TaPLDß1* | *tapldb1-ad-11* | *tapldb1-147-d* | *tapldb1-334-a* |
| *TaPLDß1* | *tapldb1-ad-12* | *tapldb1-147-d* | *tapldb1-334-a* |
| *TaPLDß1* | *tapldb1-ad-13* | *tapldb1-147-d* | *tapldb1-334-a* |
| *TaPLDß1* | *tapldb1-ad-14* | *tapldb1-147-d* | *tapldb1-334-a* |
| *TaPLDß1* | *tapldb1-ad-15* | *tapldb1-147-d* | *tapldb1-334-a* |
| *TaPLDß1* | *tapldb1-ad-16* | *tapldb1-147-d* | *tapldb1-334-a* |
| *TaPLDß1* | *tapldb1-ab-1* | *tapldb1-855-b* | *tapldb1-781-a* |
| *TaPLDß1* | *tapldb1-ab-2* | *tapldb1-855-b* | *tapldb1-781-a* |
| *TaPLDß1* | *tapldb1-ab-3* | *tapldb1-855-b* | *tapldb1-781-a* |
| *TaPLDß1* | *tapldb1-ab-4* | *tapldb1-855-b* | *tapldb1-781-a* |

**Table E.** Expected genotypic ratios as detected by the probe screen of F2 progeny used in this study. Upper case indicates intact homoeologue and lower case indicates deleted homoeologue. Index is provided for reference to Tables 1 and 2. For example, Index 1 refers to the expected genotypic ratio as detected by the probe screen within F2 progeny from a cross between an A-deletion primary mutant and a B-deletion primary mutant.

| **Population** | **Index** | **ABD** | **aBD** | **AbD** | **ABd** | **abD** | **aBd** | **Abd** | **abd** |
| --- | --- | --- | --- | --- | --- | --- | --- | --- | --- |
| F2 from AaBbDD | 1 | 56.3 | 18.8 | 18.8 | - | 6.3 | - | - | - |
| F2 from AaBBDd | 2 | 56.3 | 18.8 | - | 18.8 | - | 6.3 | - | - |
| F2 from AABbDd | 3 | 56.3 | - | 18.8 | 18.8 | - | - | 6.3 | - |
| F2 from AaBbdd | 4 | - | - | - | 56.3 | - | 18.8 | 18.8 | 6.3 |
| F2 from AaBbDd | 5 | 42.2 | 14.1 | 14.1 | 14.1 | 4.7 | 4.7 | 4.7 | 1.6 |

**Table F. Intactness of wheat homologues of genes flanking *BdPFT1* in control and mutant lines.** A green cell containing a ✓indicates that the gene is intact. A red cell containing an X indicates that the gene is deleted. A white cell containing a ? indicates that homoeologue-specific SNPs could not be identified for that homoeologue and intactness is unknown.

|  |  |  | **Wild type** | | | | **A genome** | | | | | **B genome** | | **D genome** | | | |
| --- | --- | --- | --- | --- | --- | --- | --- | --- | --- | --- | --- | --- | --- | --- | --- | --- | --- |
| **Brachypodium gene** | **Wheat EST** | **Relative to *BdPFT1*** | **Chara 1** | **Chara 2** | **CS 1** | **CS 2** | ***tapft1-616-a* 1** | ***tapft1-616-a* 2** | ***tapft1-85-a*** | **5AL-14** | **N5AT5B** | ***tapft1-157-b*** | **N5BT5A** | ***tapft1-723-d*** | ***tapft1-757-d*** | ***tapft1-734-d*** | **N5DT5A** |
| Bradi4g26880 | CK208423 | Up 1 Mb | ✔ | ✔ | ✔ | ✔ | ✔ | ✔ | ✔ | **X** | **X** | **?** | **?** | ✔ | **X** | ✔ | **X** |
| Bradi4g27120 | CD916847 | Up 700 kb | ✔ | ✔ | ✔ | ✔ | ✔ | ✔ | ✔ | **X** | **X** | ✔ | **X** | **X** | **X** | ✔ | **X** |
| Bradi4g27340 | CJ874674 | Up 400 kb | ✔ | ✔ | ✔ | ✔ | **X** | **X** | **X** | **X** | **X** | **X** | **X** | ✔ | **X** | ✔ | **X** |
| Bradi4g27490 | CK162425 | Up 200 kb | ✔ | ✔ | ✔ | ✔ | **X** | **X** | **X** | **X** | **X** | **?** | **?** | ✔ | ✔ | ✔ | **X** |
| Bradi4g27610 | HX155210 | Up 100 kb | ✔ | ✔ | ✔ | ✔ | **X** | **X** | **X** | **X** | **X** | **X** | **X** | **X** | **X** | ✔ | **X** |
| Bradi4g27720 | CJ547672 | Up 50 kb | ✔ | ✔ | ✔ | ✔ | **X** | **X** | **X** | **X** | **X** | **?** | **?** | **X** | **X** | ✔ | **X** |
| Bradi4g27740 | TA96658_4565 | Up adjacent | ✔ | ✔ | ✔ | ✔ | **?** | **?** | **?** | **?** | **?** | **X** | **X** | **X** | **X** | ✔ | **X** |
| Bradi4g27760 | EU714979 | Down adjacent | ✔ | ✔ | ✔ | ✔ | **X** | **X** | **X** | **X** | **X** | **X** | **X** | **X** | **X** | **X** | **X** |
| Bradi4g27810 | HX128956 | Down 100 kb | ✔ | ✔ | ✔ | ✔ | **X** | **X** | **X** | **X** | **X** | **X** | **X** | **X** | **X** | **X** | **X** |
| Bradi4g27880 | CJ617348 | Down 200 kb | ✔ | ✔ | ✔ | ✔ | ✔ | ✔ | **X** | **X** | **X** | ✔ | **X** | **X** | **X** | ✔ | **X** |
| Bradi4g28040 | CJ825949 | Down 400 kb | ✔ | ✔ | ✔ | ✔ | ✔ | ✔ | ✔ | **X** | **X** | ✔ | **X** | ✔ | **X** | ✔ | **X** |
| Bradi4g28150 | CJ690351 | Down 500 kb | ✔ | ✔ | ✔ | ✔ | ✔ | ✔ | ✔ | **X** | **X** | ✔ | **X** | ✔ | **X** | ✔ | **X** |
| Bradi4g28580 | BQ161472 | Down 1 Mb | ✔ | ✔ | ✔ | ✔ | ✔ | ✔ | ✔ | **X** | **X** | ✔ | **X** | ✔ | **X** | ✔ | **X** |
| Bradi4g29060 | CJ675064 | Down 1.5 Mb | ✔ | ✔ | ✔ | ✔ | ✔ | ✔ | ✔ | **X** | **X** | ✔ | **X** | ✔ | **X** | ✔ | **X** |
| Bradi4g29550 | CJ534934 | Down 2 Mb | ✔ | ✔ | ✔ | ✔ | ✔ | ✔ | ✔ | **X** | **X** | ✔ | **X** | ✔ | **X** | ✔ | **X** |

**Table G**. Assessment of conservation of synteny of genes flanking *PFT1*, targeted for the amplicon sequencing approach to assess deletion size in wheat. Under ‘5AL homoeologue’, ‘5BL homoeologue’, and ‘5DL homoeologue’, Yes/No indicates whether or not a homoeologue was identified within the CSS on that chromosome arm by *in silico* analysis; ‘?’ indicates a lack of confidence. Under ‘Translocated *in vitro*’, Yes/No indicates whether or not a translocation is indicated by the amplicon sequencing approach. Under ‘Translocated *in silico*’, Yes/No indicates whether, overall, a translocation is indicated by *in silico* analysis; ‘?’ indicates a lack of confidence.

|  | **Brachypodium gene** | **5AL homoeologue** | **5BL homoeologue** | **5DL homoeologue** | **Translocated *in vitro*** | **Translocated *in silico*** |
| --- | --- | --- | --- | --- | --- | --- |
| Up 2 Mb | Bradi4g25620 | No | No | No | Yes | Yes |
| Up 1.5 Mb | Bradi4g26150 | No | No | No | Yes | Yes |
| Up 1 Mb | Bradi4g26877 | ? | ? | ? | No | ? |
| Up 700 kb | Bradi4g27117 | Yes | Yes | Yes | No | No |
| Up 400 kb | Bradi4g27334 | Yes | Yes | Yes | No | No |
| Up 200 kb | Bradi4g27490 | Yes | Yes | Yes | No | No |
| Up 100 kb | Bradi4g27607 | Yes | Yes | Yes | No | No |
| Up 50 kb | Bradi4g27720 | Yes | Yes | Yes | No | No |
| Up adjacent | Bradi4g27740 | Yes | Yes | Yes | No | No |
| Down adjacent | Bradi4g27760 | Yes | Yes | Yes | No | No |
| Down 50 kb | Bradi4g27777 | No | No | ? | Yes | ? |
| Down 100 kb | Bradi4g27810 | ? | ? | Yes | No | No |
| Down 200 kb | Bradi4g27880 | Yes | Yes | Yes | No | No |
| Down 400 kb | Bradi4g28040 | Yes | Yes | Yes | No | No |
| Down 500 kb | Bradi4g28150 | Yes | Yes | Yes | No | No |
| Down 700 kb | Bradi4g28310 | No | No | No | Yes | Yes |
| Down 1 Mb | Bradi4g28580 | Yes | Yes | Yes | No | No |
| Down 1.5 Mb | Bradi4g29060 | Yes | Yes | Yes | No | No |
| Down 2 Mb | Bradi4g29547 | Yes | Yes | Yes | No | No |

**Table H.** Primers used for amplicon sequencing approach to assess the size of deletions removing *TaPFT1*.

| **Sequence Name** | **Sequence (5' - 3')** |
| --- | --- |
| F1_Up_50kb | GGT GAT TGA GTT CAT GCA GCA GA |
| F2_Up_50kb | TGC CCA GCA CCA TGT TCA TT |
| R1_Up_50kb | CAA CAA CAC CTC GAC GAC CTC T |
| R2_Up_50kb | CTC CAG GTC TGC CGA GGA TT |
| F1_Down_50kb | GTC AGA CTA CCG AAG ATT GC |
| F2_Down_50kb | ATG GTG AAG GCG TCA AGG AA |
| R1_Down_50kb | GAT TAT TCT GGG CCA TGA TAT |
| R2_Down_50kb | TTT TAG ACT TTT GTA GCA GGA C |
| F1_Up_adjacent | CAG TCC GGT TTC ATG GAG CT |
| F2_Up_adjacent | GGA CAA GCA GTC CGG TTT CA |
| R1_Up_adjacent | AAC TCC AAG GGC AGT AAG CA |
| R2_Up_adjacent | CCG GGA ATA TCA AAT GGT AAA |
| F1_Down_adjacent | CAC GAG CAG CCA CTG AGC TA |
| F2_Down_adjacent | AGA TCG ACA AAG CAC GAG CAG |
| R1_Down_adjacent | CAG CAC TTG AGC CTC AGG AA |
| R2_Down_adjacent | TAG CAG AAG TGG CGA TGG AA |
| F1_Up_100kb | GAA TGA GCC TGT CTC CCA GAG |
| F2_Up_100kb | TCA AGG TGG AAT GAG CCT GTC |
| R1_Up_100kb | CGA ACT GCT CCG AGA CGA TC |
| R2_Up_100kb | TTC TCC AGC AGG GTG TTG AT |
| F1_Down_100kb | GAG CCT TTT CAC CGC TTC AT |
| F2_Down_100kb | CTG GAT AAT ACT TCT CCT CCT TGC |
| R1_Down_100kb | AGA AAT CAG GAC GTG GTC GG |
| R2_Down_100kb | AAC TCC ATC CTT AAT TGC ATC AG |
| F1_Up_200kb | CGG TTC TAG TAT CCG CGT TCT |
| F2_Up_200kb | TGG CTT GCC AAT CTC CAT TA |
| R1_Up_200kb | CTC TGT ATG GAT CTC CAG CAT C |
| R2_Up_200kb | CCT CTG GAT TTC AAC TCC TCC |
| F1_Down_200kb | TGG TCC TTC ACT GTG AGT TCA A |
| F2_Down_200kb | CAC CCA AGA ATG ATG AAA TGG |
| R1_Down_200kb | ACA AAG CCA TCT TTC CAC AAG |
| R2_Down_200kb | GGT GAA GGT GCG GTG GCT GA |
| F1_Up_400kb | GGA CTT GGC TTG TCA GGA AT |
| F2_Up_400kb | ACG TTG GGT TCA TCA TCT TC |
| R1_Up_400kb | TTA ACC AAG TAG GCA CAA GAA A |
| R2_Up_400kb | CGA CAA TGG CTG TGG TAG CT |
| F1_Down_400kb | TGG ATA CCT TGG TGA AGA CCC |
| F2_Down_400kb | ACC TTA TGC CCT ACG TTC AGC |
| R1_Down_400kb | CAG CAA GAT CAA GCA CAT GAA TA |
| R2_Down_400kb | AGT GCA GCA ATA TGC CCC TC |
| F1_Up_500kb | AAA TGA TAA GGA GGT ACA CGA AC |
| F2_Up_500kb | TAC ACG AAC CCA AAG ACC AG |
| R1_Up_500kb | CCT TTC GAC TCC GAA CTT CTT |
| R2_Up_500kb | TCT CAT TCA AGG GAT CAA ACC |
| F1_Down_500kb | AGG AGT AGT AGC AGC AGC TTT CTT GG |
| F2_Down_500kb | GCT TTC TTG GAC CGG ACA TCA |
| R1_Down_500kb | TGG TTG CCG TGG ACT CGT AA |
| R2_Down_500kb | CCG ACT TCC TCA CAA CCC TTT ACT T |
| F1_Up_700kb | ATG GGA GAC AGT TCG AGG ATA T |
| F2_Up_700kb | AAA GGA TGC TCA GGC TTC AA |
| R1_Up_700kb | AAT GCT ACT GGA TCT GAG GGT G |
| R2_Up_700kb | GTG GTG GCA TTT CGT CTT TT |
| F1_Down_700KB | TAA CGA AAG TCG CCA TTC TAG |
| F2_Down_700KB | TGG TGG GCT TAT GGG TTC TG |
| R1_Down_700KB | TTG CTG CTT CAA CTT CAC ATT CTC |
| R2_Down_700KB | CGG TCT TGG GAC TTT GTT TT |
| F1_Up_1Mb | GTT GTT GCT TAC AGG CGT GG |
| F2_Up_1Mb | GCG TGG ACA ATG TGA TGA TG |
| R1_Up_1Mb | ACA GCA TCT TCT TCA ACA CG |
| R2_Up_1Mb | GCC CTA TCC ATC TTC GCA TA |
| F1_Down_1Mb | CAA TCA CAG AGG CCA TGC TA |
| F2_Down_1Mb | ATT TCC AAA CCT CCC TAA CAT |
| R1_Down_1Mb | CCT GAT GGC TGA GAC TGT TTA T |
| R2_Down_1Mb | GGA GAT GCT GTC CAG GAT AAA |
| F1_Up_1.5Mb | AGT GTT CGT GCT GTC TGA CTT CG |
| F2_Up_1.5Mb | AGG ATC TCA TTG CGC CAT ACC |
| R1_Up_1.5Mb | TTC CCT GGC TGG CTT TGC TG |
| R2_Up_1.5Mb | GAG GAA CGG ACA AAG ATG GAG |
| F1_Down_1.5Mb | CAC TAC CAA CAG ACC TGC AAT C |
| F2_Down_1.5Mb | GCA ATC GGA GGA CCA TTT AA |
| R1_Down_1.5Mb | GAG CTG GTG AAT CAC GTT GAG GAT |
| R2_Down_1.5Mb | TTC TTG TGC CAT CTG TCT TAT TG |
| F1_Up_2Mb | TCC AGT TTC AGA GTC CCT AGA CAA |
| F2_Up_2Mb | TCC AGA CTG GGA TAG CAG ATT C |
| R1_Up_2Mb | AGG ATC AGG TGG TCC AAA GG |
| R2_Up_2Mb | AAG AAT GAC AAG GAT CAG GTG G |
| F1_Down_2Mb | GGC TTC CCG TTC AAC TTT GG |
| F2_Down_2Mb | AAG AAC TTT GCT TTT GTG GG |
| R1_Down_2Mb | AGC CCT TGA TAG ACA TGA AGA CCA G |
| R2_Down_2Mb | GAG GGG ATC TGA GGC TCC TT |

**Table I.** MEGABLAST matches (E-value = 0) of candidate gene sequence obtained from wheat cv. Chara against Chinese Spring ‘Genome Survey Sequence’.

| **Query** | **Target** | **Identity** | **Length** | **E-value** |
| --- | --- | --- | --- | --- |
| TaPLDB1 | Chr1BL_3831514 | 100 | 613 | 0 |
| TaPLDB1 | Chr1DL_648118 | 95.32 | 619 | 0 |
| TaPLDB1 | Chr1AL_3845214 | 93.54 | 619 | 0 |
| TaWRKY11 | Chr2AL_6367445 | 98.3 | 943 | 0 |
| TaWRKY11 | Chr2BL_8031991 | 95.59 | 952 | 0 |
| TaPFT1 | Chr5BL_10841930 | 100 | 921 | 0 |
| TaPFT1 | Chr5DL_4488901 | 94.39 | 927 | 0 |
| TaPFT1 | Chr5AL_2461897 | 93.45 | 932 | 0 |
