# Supplementary material for: An Assessment of Heavy Ion Irradiation Mutagenesis for Reverse Genetics in Wheat (Triticum aestivum L.)
Source: PLoS One. 2015 Feb 26;10(2):e0117369. doi: 10.1371/journal.pone.0117369 (PMC4342231; doi:10.1371/journal.pone.0117369)
Supplement: S2 File — (DOCX) [file pone.0117369.s012.docx]

**Supplementary Material and Methods**

***CAPS genotyping***

For CAPS screening to validate *TaWRKY11* deletions, gene-specific *TaWRKY11* primers were designed using Primer3 (<http://bioinfo.ut.ee/primer3-0.4.0/primer3/input.htm>) targeting a region conserved between all homoeologues. SNP2CAPS (<http://pgrc.ipk-gatersleben.de/snp2caps/>) was used to identify a restriction digest that would produce homoeologue-specific fragments; digestion with ScrFI was selected which was predicted to yield fragments of 152, 123 and 105 bp for the B, D and A homoeologues, respectively. PCR to amplify *TaWRKY11* fragments was performed using 5 µL High Fidelity Phusion PCR Buffer (New England BioLabs, Inc.), 0.5 µL 10 mM dNTPs, 0.75 µL DMSO, 0.25 µL Phusion proofreading DNA polymerase, 1 uL 10 µM forward and reverse primers, 2 µL ~ 50 ng/µL DNA template and 15.5 µL DNase-free water. Cycling conditions were: hot start at 98ºC; 1 min at 98ºC; 15 cycles with -1ºC annealing temperature per cycle starting with 98ºC for 15s, 70ºC for 15s, 72ºC for 30s; 25 cycles of 98ºC for 15s, 58ºC for 15s, 72ºC for 30s; 72ºC for 5 mins; hold at 22ºC. PCR products were then digested at 37ºC overnight using ScrFI and products were visualized on a 2.5% agarose gel stained with GelRed (Biotium, Inc., Hayward, CA 94545, USA) run at 75V for 90 mins.

***PCR genotyping***

For agarose gel-based validation of *TaPLDß1* hexas, *TaPLDß1* primers designed for the high-throughput probe screen, were combined with primers targeting the wheat gene *TaNFXL1* as a control*.* Design of both primer sets is described in [16]. Duplex PCR was performed using the conditions described for amplification of *TaWRKY11* PCR products for CAPS screening, as above. PCR products were visualized on a 1.5% agarose gel stained with GelRed (Biotium, Inc., Hayward, CA 94545, USA) run at 95V for 65 mins.

***In silico* assessment of chromosomal location of *PFT1* flanking genes**

As outlined in Results, for genes in the region from 1Mb to 2Mb ‘Up’ of *BdPFT1*, macrosynteny appears to have been lost. To assess whether there was a consistent pattern of translocation of these genes, we assessed the chromosomal location of the best match within the CSS for translocated genes in this region, using data from analysis described in Material and Methods. For 83 of the 99 genes, the best match occurred within the chromosome 4 series. Best matches identified on the A subgenome were predominantly located on chromosome 4AL, while best matches identified on the B and D subgenomes were predominantly located on 4BS and 4DS, respectively. For all genes in this region, we then assessed whether the wheat homoeologues were likely to be identified on 4AL, 4BS and 4DS, using the logic described for our initial analysis of conservation of synteny in the *TaPFT1* region (Material and Methods) implemented using a custom Python script (PFT1_flank_CSS_hits_step4.py).

**Supplementary Results**

***In silico* assessment of chromosomal locations of *TaPFT1*, *TaPLDß1*, and *TaWRKY11***

Gene sequence of *TaPFT1*, *TaPLDß1* and *TaWRKY11* used for the design of the high-throughput deletion screening assays was used for a MEGABLAST search of the CSS, as described in Results. Matches with an E-value of ‘0’, highly likely to be homoeologues or gene duplications, were targeted. Matches to *TaPFT1* sequence were identified on the long arms of the three group 5 chromosomes (Table S9) and matches to *TaPLDß1* were identified on chromosomes on the long arms of the three group 1 chromosomes (Table S9), consistent with results of screening of nullisomic-tetrasomic lines using the high-throughput method performed here and in Fitzgerald *et al*. (2010). Matches to *TaWRKY11* were identified on chromosomes 2AL and 2BL (Table S9). The corresponding *TaWRKY11* sequence was not identified on the 2D chromosome sequence in the CSS, despite screening of nullisomic-tetrasomic lines suggesting that a homoeologous copy of the gene resides on chromosome 2D. The degree of completeness of the CSS has been estimated at 61% overall [27]; therefore, although the coverage is likely to be higher for gene regions, the most likely explanation for this is that no sequence coverage of the *TaWRKY11-D* gene region is present within the CSS. The *TaWRKY11* sequence on chromosome 2A possessed sequence identical to the region targeted by the WRKY11prbVIC probe (Figure S9), indicating that this probe targeted the *TaWRKY11-A* homoeologue (Table S3) and complementing results of nullisomic-tetrasomic screening performed as described above. Furthermore, the *TaWRKY11* sequence on chromosome 2B (Figure S9) possessed sequence identical to the regions targeted by the WRKY11prbNED probe, as predicted by nullisomic-tetrasomic screening.

***Gel-based validation of tetras and hexas***

A subset of *TaPFT1* and *TaWRKY11* tetras confirmed using the probe screening method were validated independently using agarose gel-based CAPS screening. For validation of the genotype of *TaPFT1* tetras identified using the high-throughput probe-based method, the homoeologue-specific CAPS screen described in [16] was used (e.g. Figure S10). In all cases, tetras identified by the high-throughput method and confirmed at F3 stage were confirmed using the CAPS method. For validation of *TaWRKY11* tetras, a *TaWRKY11* homoeologue-specific CAPS screen was designed (Supplementary Materials and Methods, above). As for *TaPFT1*, in all cases, *TaWRKY11* tetras identified by the high-throughput method and confirmed at F3 stage were also confirmed using the CAPS method (e.g. Figure S10). For independent validation of all *TaPLDB1* hexas, a duplex PCR incorporating primers targeting all homoeologues of *TaPLDß1* and control primers was used (Supplementary Materials and Methods, above). For one line identified as a hexa using the high-throughput screen, the presence of at least one *TaPLDß1* homoeologue was indicated by the duplex PCR. In all other cases, the gel-based screen confirmed the hexa status of these lines (e.g. Figure S10).

**In silico *assessment of chromosomal location of* PFT1 *flanking genes***

For the region from 1Mb to 2Mb up from *BdPFT1*, macrosynteny appears to have been lost between wheat and Brachypodium. For most genes in this region, the best match within the CSS was identified within the chromosome 4 series, on 4AL, 4BS, or 4DS. Therefore, we assessed whether a putative homoeologue was present on 4AL, 4BS, and 4DS for all translocated genes within this region (Supplementary Materials and Methods, above). As outlined in File S6, for the majority of genes this was found to be the case.
